# Metabolomics and lipidomics in the differentiation of CIN 2 and CIN 3: an exploratory pilot study

**DOI:** 10.1007/s11306-026-02555-w

**Published:** 2026-11-13

**Authors:** Luana Quadros de Souza Leão, Giovanna Melo Marques, Adriano de Souza Carolino, Ana Carolina Rosa da Silva, Sofia Angiole Cavalcante, Elzalina Ribeiro Soares, Jonas Balan de Padua, Anderson Ferreira Gonçalves, Marina Amaral Alves, Rafael Garrett, Priscila Ferreira de Aquino

**Affiliations:** 1 Postgraduate Program in Chemistry (PPGQ), Federal University of Amazonas (UFAM), Manaus, AM Brazil; 2 Leonidas and Maria Deane Institute/Fiocruz Amazônia (ILMD/Fiocruz Amazônia), Manaus, AM Brazil; 3 Postgraduate Program in Materials Science and Engineering (PPGCEM), Federal University of Amazonas (UFAM), Manaus, AM Brazil; 4 Metabolomics Laboratory (LabMeta), Institute of Chemistry, LADETEC - Federal University of Rio de Janeiro (IQ/UFRJ), University City, Rio de Janeiro, RJ Brazil; 5 SPARC BioCentre, The Hospital for Sick Children, Toronto, ON Canada; 6 Department of Chemistry, Amazonas State University (UEA), Tefé, AM Brazil; 7 Amazonas State Oncology Control Center Foundation (FCECON), Manaus, AM Brazil; 8 Laboratory of Metabolomics Applied to Systems Medicine (Meta2MS), Federal University of Rio de Janeiro (IPPN/UFRJ), University City, Rio de Janeiro, RJ Brazil

**Keywords:** Cervical intraepithelial neoplasia, Metabolomics, Lipidomics, LC-HRMS

## Abstract

**Introduction:**

High-grade cervical intraepithelial neoplasia (CIN 2–3) is a precursor lesion to cervical cancer and, although it presents different probabilities of regression and progression, it is usually grouped together in screening and managed similarly. However, the mechanisms that determine its evolution are still poorly understood, and standard treatment by conization can lead to reproductive complications such as subfertility and premature birth.

**Objective:**

This study aimed to identify metabolic and lipid signatures that can distinguish between CIN 2 and CIN 3 lesions using liquid chromatography-high-resolution mass spectrometry (LC-HRMS).

**Methods:**

Tissue samples from the lesion and adjacent margin of 24 women with CIN 2–3 were subjected to metabolomic and lipidomic analysis by LC-HRMS. Multivariate statistical analyses, pathway analysis, and correlation were performed to understand these neoplasms’ development and progression and identify potential metabolites and lipids to distinguish such lesions.

**Results:**

Metabolomic and lipidomic analysis by LC-HRMS in cervical tissue identified molecular signatures with differential abundance between CIN 2 and CIN 3, highlighting patterns associated with the degree of lesion. Multivariate analyses highlighted metabolites (serine, glutamic acid, guanidine-6-monophosphate, succinic acid, mannose 6-phosphate, and taurine) and lipids (TG 49:1, TG 50:1, and TG 52:2) as the main contributors to the discrimination between the groups, suggesting relevant biological alterations in high-grade cervical lesions.

**Conclusion:**

Combination metabolomic and lipidomic approach enabled the identification of metabolites and lipids potentially involved in the pathogenesis of intraepithelial neoplasia (CINs). In addition, these identified metabolite and lipid panels are promising as complementary tools for future screening strategies for these lesions.

**Graphic Abstract:**

**Figure:**
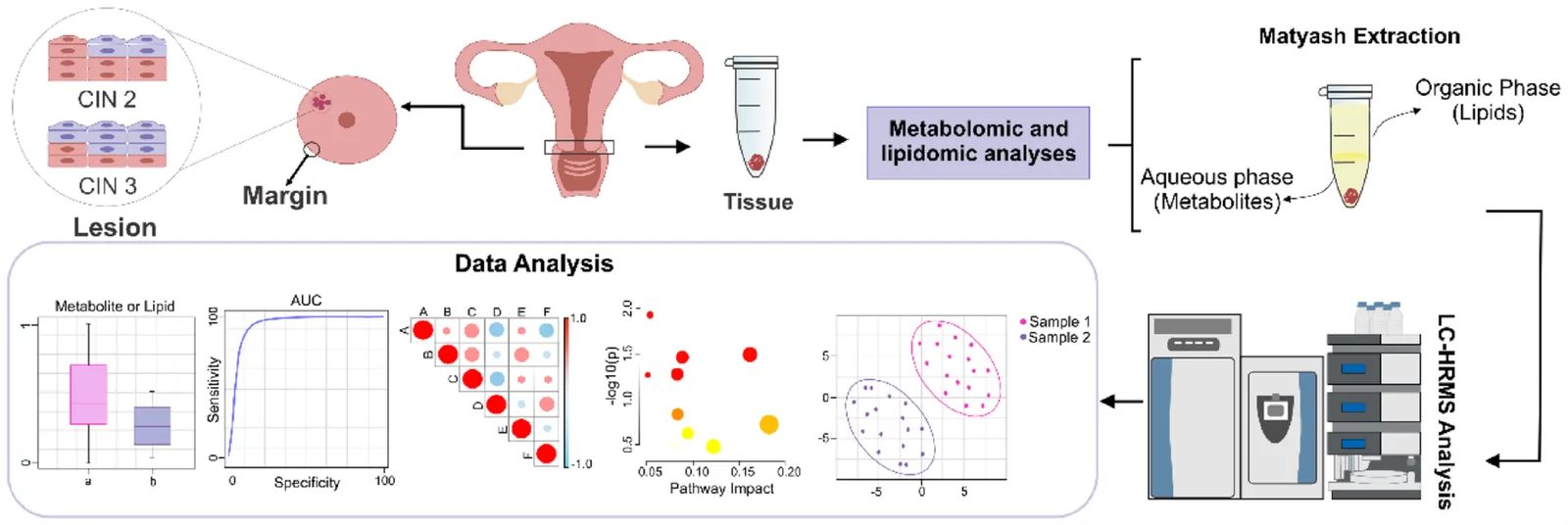

**Supplementary Information:**

The online version contains supplementary material available at https://doi.org/10.1007/s11306-026-02555-w.

## Introduction

Cervical cancer (CC) is a tumor that has a high chance of being cured when diagnosed and treated early. Despite this, the incidence and mortality rates for this neoplasm remain high, being the fourth most common among women in terms of incidence and mortality worldwide (International Agency for Research on Cancer, 2025).

The development of this disease is preceded by lesions called cervical intraepithelial neoplasias (CINs). These neoplasias can be classified into grades according to the thickness of the cervical epithelium affected by the disease. They can be low-grade (CIN 1) or high-grade (CIN 2 and CIN 3) (Loopik et al., 2021; Östör, 1993). Specifically, high-grade lesions are usually treated with a conization procedure such as loop electrosurgical excision (LEEP) (WHO, 2024). Despite being a procedure for preventing cervical cancer, LEEP can present risks related to reproductive complications, such as an increased risk of subfertility and premature birth (Athanasiou et al., 2022; Bergqvist et al., 2025). In developed countries, some guidelines recommend conservative treatment, such as active surveillance of women with CIN 2 (Macdonald et al., 2018; Wright et al., 2007). Because it is an intermediate lesion, CIN 2 has a higher probability of regression when compared to CIN 3. In addition, studies show that CIN 2 lesions regress spontaneously in approximately 60% of untreated women (Brun et al., 2021; Lycke et al., 2023).

However, distinguishing between a CIN 2 lesion and a CIN 3 lesion can be complex. In fact, this differentiation depends on the histological analysis that several variables, including sample quality, histological overlap, and subjectivity in interpretation, can influence (Carreon et al. 2007; Del Pino et al. 2021; Yang et al. 2012). Therefore, the need remains for strategies that reduce diagnostic uncertainty and deepen the understanding of the biological mechanisms involved in cervical neoplasia, especially in the context of high-grade lesions. In this scenario, approaches such as metabolomics and lipidomics have been the target of studies aimed at characterizing metabolic and lipid signatures associated with cervical lesions and identifying candidate markers that may, in the future, complement histopathological evaluation and contribute to risk stratification (Khan et al. 2019; Nam et al. 2021; Starodubtseva et al. 2022; Tokareva et al. 2022). Mainly focusing on precursor lesions of CC, a study carried out by Nuer-Allornuvor et al. (2024) identified in plasma samples the metabolites 2-methyl-1-propylamine and estrone as potential targets for differentiation of the early stages of CIN (CIN 1 and CIN 2) in healthy women and patients with invasive carcinoma (Nuer-Allornuvor et al. 2024). Furthermore, a study by Cheng et al. (2020) identified a panel of lipids from the phosphatidylcholine and phosphatidylethanolamine classes as a promising serum biomarker to discriminate early-stage CC from squamous intraepithelial lesions and healthy subjects (Cheng et al. 2020).

Therefore, the combination of these omics may provide a comprehensive perspective on the metabolic landscape relevant to diseases (Yan et al., 2024). This study aimed to identify metabolic and lipid signatures that can distinguish between CIN 2 and CIN 3 lesions using liquid chromatography-high resolution mass spectrometry (LC-HRMS), which may be helpful in developing new approaches for screening and early detection of this disease or suggest metabolite associations with the possible progression of these neoplasms. These advances may impact the treatment and survival of patients affected by high-grade CIN.

## Materials and methods

### Patients

Women with a pathologically confirmed diagnosis of cervical intraepithelial neoplasia (CIN) grades 2 or 3 were selected for participation in the study. All participants signed an informed consent form, and this project was approved by the Ethics Committee of the Oncology Control Foundation Center of Amazonas State (CEP/FCECON: 71342417.4.3002.5020-CAAE; Manaus, Amazonas). In total, twenty-four women (twelve patients for each group) were selected according to the following criteria: (i) Women over 18 years of age who had been referred for surgical conization by the clinical team of the CECON Foundation and with a pre- and postoperative histopathological report of high-grade intraepithelial neoplasia (CIN 2 or CIN 3); (ii) Women who had not undergone cervical treatment previously. Pregnant patients and those who presented with the presence or history of any type of carcinoma at the time of collection were excluded from the study.

### Sample collection and preparation

In total, 48 cervical tissue samples were collected during the conization surgical procedure, 24 samples from the lesion and 24 samples from the adjacent margin. Three distinct tissue masses were obtained for analysis, considering the collected material available. Thus, 10, 5, and 2.5 mg of tissue samples were weighed and subjected to freeze-thaw maceration cycles, consisting of a cycle of freezing in liquid nitrogen (10 min) and thawing in the refrigerator − 20 °C (10 min). This process was repeated three times. It’s worth noting that the tissue mass was normalized by resuspension and injection volume of the samples.

Subsequently, these samples were subjected to a modified biphasic Matyash extraction (Matyash et al., 2008) comprising MTBE, methanol, and water (10:3:2.5; v/v/v). For extraction, 300 µL of MeOH was added to the tissue samples and vortexed for 1.0 min. Then, 1000 µL of MTBE was added, and the samples were subjected to an ultrasonic bath for 15 min, followed by the addition of 250 µL of water. Afterwards, the samples were incubated for 15 min at -20 °C and centrifuged for 10 min at 9000 rpm. The organic phase (lipids) and the aqueous phase (metabolites) were collected, dried under a nitrogen flow at 40 °C, and stored at − 20 °C until analysis.

In addition, a quality control (QC) sample was prepared by mixing equal volumes of each sample from all groups after extraction. The QC was analyzed throughout the sequence at every ten sample injections to monitor the stability and performance of the liquid chromatography-mass spectrometry system.

### LC-HRMS data acquisition

The LC-HRMS/MS analyses were performed using a Dionex Ultimate 3000 liquid chromatography coupled to a hybrid Quadrupole-Orbitrap high-resolution mass spectrometer (Thermo Scientific Q-Exactive Plus) with an electrospray ionization (ESI) source operating in positive and negative mode for metabolomics and in positive mode for lipidomics (Alves et al., 2021; Silva et al., 2020). The samples were analyzed in random order, and a blank sample was injected at the beginning and end of the batch. For metabolomic analysis, the dried samples were resuspended in acetonitrile/water solution (1:1; v/v). The chromatographic separation was performed in a Waters Xbridge Amide column (150 × 4.6 mm, 3.5 μm) maintained at 40 °C. Sample injection was performed with a volume of 5 µL, and the total analysis duration was 28 min. For the mobile phases, Milli-Q water containing 10 mM ammonium acetate and 0.04% ammonium hydroxide (mobile phase A) and 95% acetonitrile containing 10 mM ammonium acetate, 0.04% ammonium hydroxide and 5% Milli-Q water (mobile phase B) were used. Gradient elution occurred at a flow rate of 0.5 mL/min. Mass spectrometry conditions were: spray voltage of 3.6 kV, ion transfer capillary temperature of 300 °C, sheath and auxiliary gases at 50 arbitrary units (a.u.), normalized collision energy (NCE) of 15–40 eV. Data were acquired in full ion scan in the *m/z* range of 100–1000 at a resolution of 35,000, followed by data-dependent acquisition (ddMS2) using a resolution of 17,500.

For lipidomic analysis, organic phase from the biphasic extraction was resuspended in 50 µL of isopropanol: ACN: H_2_O (2:1:1; v/v). Chromatographic separation was performed on a Waters XSelect CSH C_18_ column (150 × 2.1 mm; 2.5 μm) with temperature maintained at 45 °C in gradient elution mode using ACN: H_2_O (60:40, v/v) as mobile phase A and isopropanol: ACN (90:10, v/v) as mobile phase B, both with 0.1% formic acid and 10 mM ammonium formate. Sample injection was performed with a volume of 5 µL, and the total analysis duration was 25 min. The solvent flow rate was 0.4 mL/min. Mass spectrometry conditions were: spray voltage of 3.9 kV, ion transfer capillary temperature of 320 °C, sheath and auxiliary gases 45 and 20 au, respectively, and NCE of 20 eV. Data were acquired in full scan in the *m/z* range 120–1200 at a resolution of 35,000, followed by the ddMS2 experiment at a resolution of 17,500.

### Data processing and annotation of metabolites and lipids

Raw data were analyzed by Thermo Xcalibur v3.0 software and preprocessed by a metabolomics and lipidomics workflow using MS-DIAL software (RIKEN, version 4.98) (Takeda et al., 2024; Tsugawa et al., 2015). This workflow included peak detection, deconvolution, chromatogram alignment, and feature annotation. The parameters used are detailed in Supplementary Material 1, Table S1.

Compound annotation was performed using MS-DIAL software, employing a standard MS/MS lipid library and a customized MS/MS metabolite database composed of the MassBank of North America and NIST 2020 libraries. For metabolomic analysis, the criteria adopted were an MS/MS similarity score greater than 80% and a maximum mass error of 5 ppm between experimental and reference ions. Furthermore, only species detected in at least 70% of the quality control (QC) samples were considered. The level of metabolite identification was assigned according to the Metabolomics Standards Initiative (MSI) guidelines (Sumner et al., 2007). Additionally, feature annotation was supplemented using the CEU Mass Mediator, the Human Metabolome Database (HMDB) (Wishart et al., 2018) and Metabolite Mass Spectral Database (Metlin) (Guijas et al., 2018) as well as the CFM-ID 4.0 platform (Gil de la Fuente et al. 2018). Further details are available in Supplementary Material 1 (Tables S2) and Supplementary Material 2 (Figures S1 and S2).

For putative lipid annotation, features were initially filtered for analytical reproducibility, retaining those detected in at least 70% of quality control (QC) samples. Putative lipid annotation was performed using the precursor ion mass-to-charge ratio (m/z), MS/MS spectra, and LC elution profiles (Silva et al., 2022). Criteria for accepting candidate annotations included an absolute mass error ≤ 5 ppm and an MS/MS similarity score > 80%. Fragmentation patterns for each lipid class are described in Table S3 of Supplementary Material 1. Nomenclature followed the LIPID MAPS classification and shorthand notation system (Liebisch et al., 2020). Annotation confidence levels were assigned according to the Metabolomics Standards Initiative (MSI) recommendations. The putative annotations are available at Table S4 of Supplementary Material 1 and Supplementary Material 2 (Figures S3).

### Statistical analysis

Multivariate statistical analysis was performed on the metabolic and lipid profile data sets independently to determine the differentially abundant metabolites and lipids present in the CIN2 and CIN3 groups. The aligned MS-DIAL data matrix was exported as a .txt file and converted to .csv for uploading into the Metaboanalyst 6.0 online platform (https://www.metaboanalyst.ca/). Data filtering was performed in which variables with relative standard deviation RSD > 30% of the grouped QCs were removed. The parameters used were median normalization, Log transformation, and auto-scaling for principal component analysis (PCA), sparse partial least-squares discriminant analysis (sPLS-DA), and orthogonal partial least-squares discriminant analysis (OPLS-DA).

The performance of the sPLS-DA models was evaluated using 5-fold cross-validation, and the optimal number of components was selected based on the lowest classification error rate. Accuracy was estimated as 1 (classification error rate), and the parameters adopted for each data matrix are presented in Table S5 of Supplementary Material 1. Specifically, in the OPLS-DA model, the adjustment capacity (R2Y) and predictability (Q2) were parameters used to evaluate the performance of the three models obtained in this study. Model robustness was further evaluated by permutation testing, and the specific parameters for each dataset are present in Table S6 of Supplementary Material 1. In parallel, univariate analysis was performed using Student’s t-test, false discovery rate (FDR) correction, and log2 fold change (log2FC) to assess the statistical significance, magnitude, and Direction of the differences between CIN2 and CIN3. Metabolites and lipids were considered statistically significant when they simultaneously showed a p-value < 0.05 and FDR < 0.05 (Supplementary Material 1, Table S7 and S8). In addition, variable importance in projection (VIP) values greater than 1 from the OPLS-DA models were considered when supported by univariate criteria, thereby allowing the selection of metabolites and lipids with the greatest impact on group discrimination. To visualize the differentially abundant patterns identified by the univariate analysis, heatmaps were constructed using only the metabolites and lipids that met the predefined univariate significance criteria (p-value < 0.05 and FDR < 0.05). Hierarchical clustering of the samples and selected metabolites/lipids was performed using Euchidean distance and Ward’s method (*ward.D2*). In the heatmap samples were identified according to the CIN2 and CIN3 groups, allowing visualization of difference in abundance profiles between the classes. Heatmaps were generated in *R* using the *pheatmap* package and are presented in Supplementary Material 2 (Figures S4 and S5 for metabolites and are lipids, respectively). Additionally, the discriminative capacity of the chosen marker candidates was evaluated using the ROC (Receiver Operating Characteristic) curve, considering only those with accuracy greater than 70%.

### Pathway, enrichment and correlation analysis

To investigate the metabolic pathways associated with differential metabolites and lipids, their HMDB IDs were inserted into the pathway analysis contained in Metaboanalyst 6.0, using the following parameters: visualization method – scatter plot; enrichment method – Fisher’s Exact Test; topology measure – relative-betweenness centrality; and pathway library, Homo sapiens (SMPDB). Similarly, enrichment analysis was performed using Metaboanalyst 6.0, with SMPDB for pathway-based analysis (Supplementary Material 1, Table S9 and S10).

The correlations between metabolites and lipids were assessed using Pearson’s correlation coefficient, using the Hmisc package in R language, considering associations with *p* ≤ 0.01 as significant. The statistically significant associations between pairs of variables were compiled in a table and are available in Supplementary Material 1, Table S11.

## Results and discussion

Specific details of the study participants are described in Table 1.

**Table 1:** General characteristics of study participants

Variables	CIN 2	CIN 3
	Mean (Min -Max)	Mean (Min -Max)
Age (years)	41.25 (25–66)	44.42 (27–67)
	f (%)	f (%)
Menstrual cycle phase		
Women of reproductive age	33	33
Postmenopausal women	17	17
Hormone use		
Hormonal contraceptives	29	42
Smoking status		
Current smoker	17	25
Never smoked	33	25

*CIN* Cervical intraepithelial neoplasia; *Min* Minimum; *Max* Maximum; (f%) = Relative Frequence

### Overview of the global metabolome and lipidome of high-grade CINs and their adjacent margins

For the metabolomic and lipidomic study, all cervical tissue samples (lesion and adjacent margin) were subjected to extraction using the Matyash method and subsequently analyzed by LC-HRMS. Multivariate analysis for metabolomics and lipidomics was performed using principal component analysis (PCA) and sparse partial least-squares discriminant analysis (sPLS-DA) on all samples, including CIN 2 (lesion and adjacent margin) and CIN 3 (lesion and adjacent margin). In the PCA, it is observed that all QC samples are clustered together, indicating good instrument performance and stability (Supplementary Material 2, Figure S6).

**Fig. 1:**
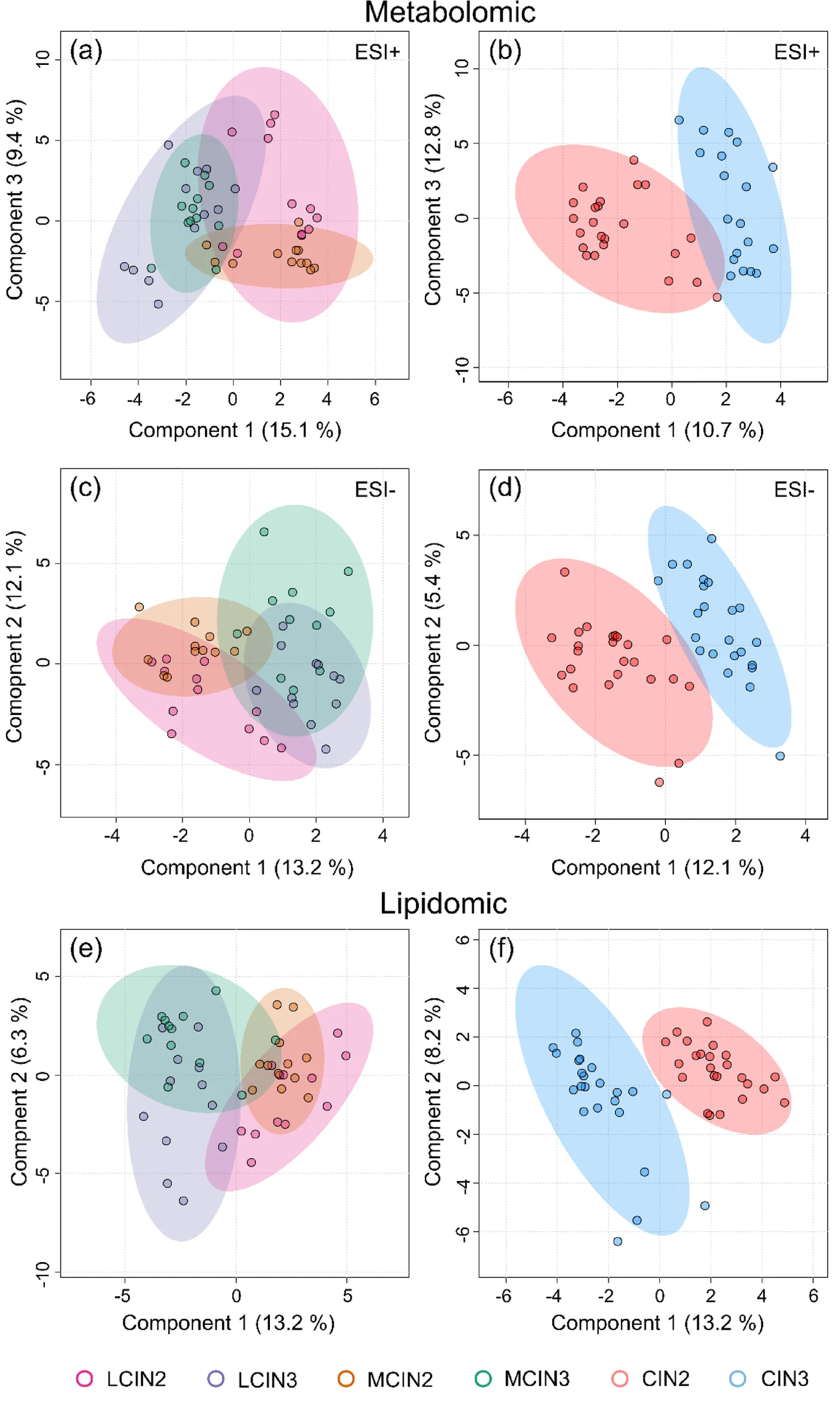
sPLS-DA of samples from the metabolomic and lipidomic study. In (**a**), (**c**) and (**e**): CIN 2 – lesion (LCIN2: pink) and margin (MCIN2: orange) and CIN 3 – lesion (LCIN3: purple) and margin (MCIN3: green). In (**b**), (**d**) and (**f**): CIN 2 – lesion and margin (CIN2: red) and CIN 3 – lesion and margin (CIN3: red)

In the sPLS-DA (Fig. 1a, c and e), a partial separation was observed between samples from the CIN 2 group (LCIN2 – lesion and MCIN2 – margin) and those from the CIN 3 group (LCIN3 – lesion and MCIN3 – margin). This distinction suggests underlying molecular differences associated with lesion severity, although the separation was not complete. Considering that all samples from the adjacent margins of this project are lesion-free according to the histopathological report, this fact is quite interesting, since the similar metabolic profiles in the lesion and margin of each group studied may present two hypotheses. The first hypothesis is that the model cannot distinguish these groups based on the selected metabolites due to the similarity of their metabolic profiles. The second hypothesis is that, although the histopathological reports confirmed that the adjacent margins were free of lesions, this assessment describes tissue morphology and does not detect molecular alterations, such as those observed in the metabolite/lipid profile. Furthermore, the adjacent margins were not considered independent controls, as they belong to the same anatomical zone as the lesion, consistent with the observed metabolic and lipid similarities. Previous reports of residual disease or recurrence of CINs at histologically free margins (Alder et al. 2020; Ding et al. 2023; Juliato et al. 2000) also reinforce the notion that the absence of a detectable lesion on histopathological examination does not exclude the possibility of subtle molecular alterations, although this interpretation should be confirmed in future studies.

Based on this pattern of similarity between margin and lesion within each CIN, it was decided to carry out the analyses by grouping the data set into two categories: CIN2, which includes lesions and margins, and CIN3, which includes lesions and margins (Fig. 1b, d, and f). After grouping, a more evident separation was observed between CIN2 and CIN3 by sPLSDA, although there was still slight overlap between the categories. We emphasize that the absence of formal adjustment for clinical covariates, such as age, menopausal status, hormone use, and smoking, represents a limitation of the study. Although these variables may influence metabolomic and lipidomic profiles, the limited sample size restricted the application of more robust adjusted models. Future studies with larger cohorts should incorporate these covariates into appropriate statistical models to assess their influence on the metabolic and lipid alterations associated with the degree of CIN.

**Fig. 2:**
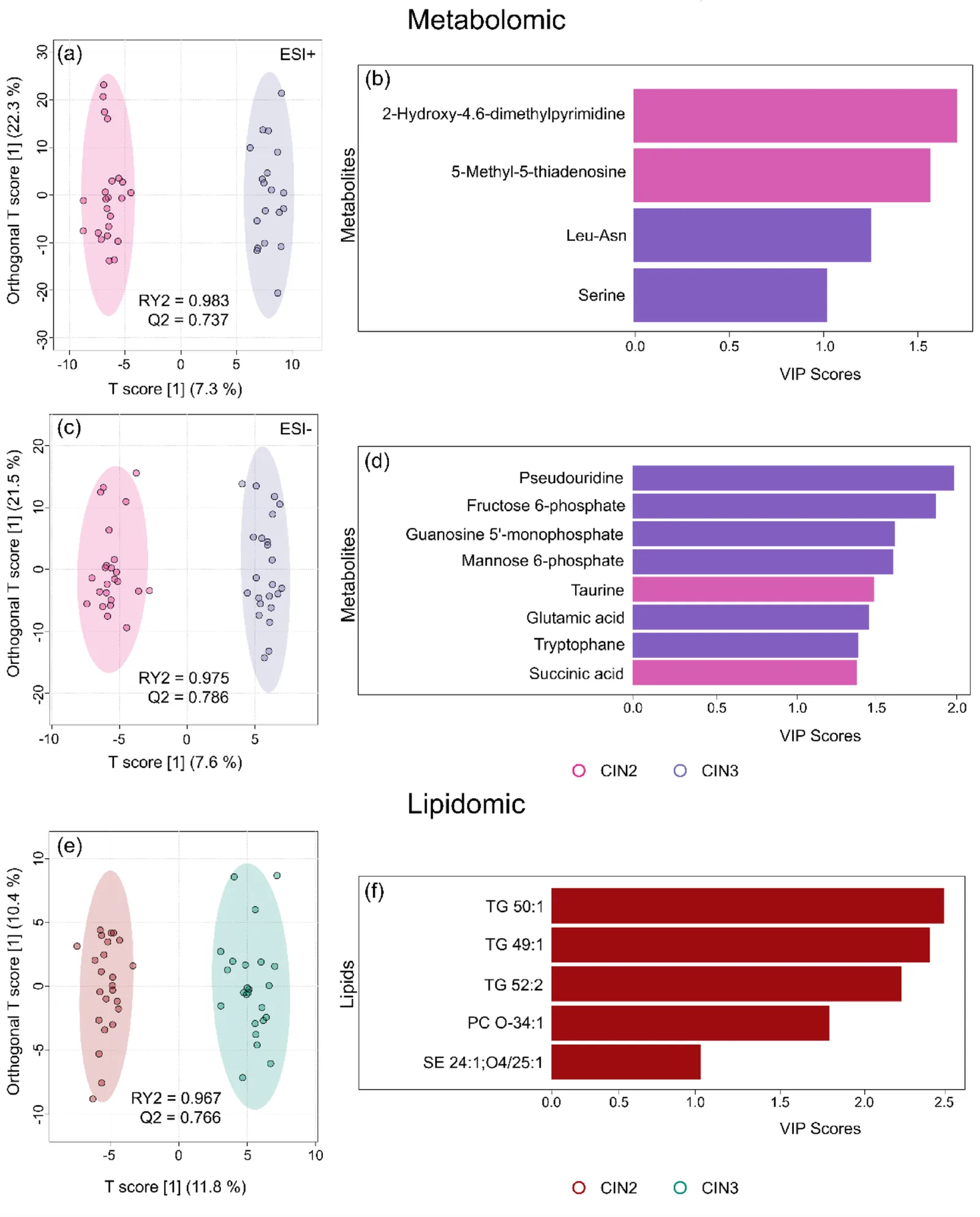
OPLS-DA of samples (letters **a**, **c**, **e**) of CIN 2 – lesion and margin (CIN2 – in pink in the metabolomic analysis; in red in the lipidomic analysis) and CIN 3 – lesion and margin (CIN3 – in purple in the metabolomic analysis; in green in the lipidomic analysis). VIP Scores containing the metabolites (letters **b**, **d**) and lipids (letter **f**) with the greatest impact on the discrimination of the CIN2 and CIN3 classes

Subsequently, this analysis was refined to maximize separation between the two categories and to assess class-related predictive variance using OPLS-DA (Fig. 2a, c, e), a model widely implemented to optimize separation in binary classification analyses. Through this analysis, it is possible to verify that there is a clear separation between the CIN 2 and CIN 3 groups, with excellent adjustment (R2Y) and predictive abilities (Q2) both in metabolomics (Fig. 2a, c) and in lipidomics (Fig. 2e). According to the influence of metabolites and lipids in the samples studied, it was possible to ascertain the impact variables contributing to the differentiation between the CIN2 and CIN3 categories displayed in OPLS-DA. A total of 4 differential metabolites were identified by comparing CIN 2 and CIN 3 in ESI+ mode (Fig. 2b) and 8 differential metabolites in ESI- mode (Fig. 2d). The 12 metabolites and 5 differential lipids most relevant to OPLS-DA analysis as potential metabolic and lipid signatures to distinguish high-grade cervical lesions are presented in Supplementary Material 1 (Tables S7 and S8).

### Metabolomic and lipidomic combination of high-grade NICs

**Fig. 3:**
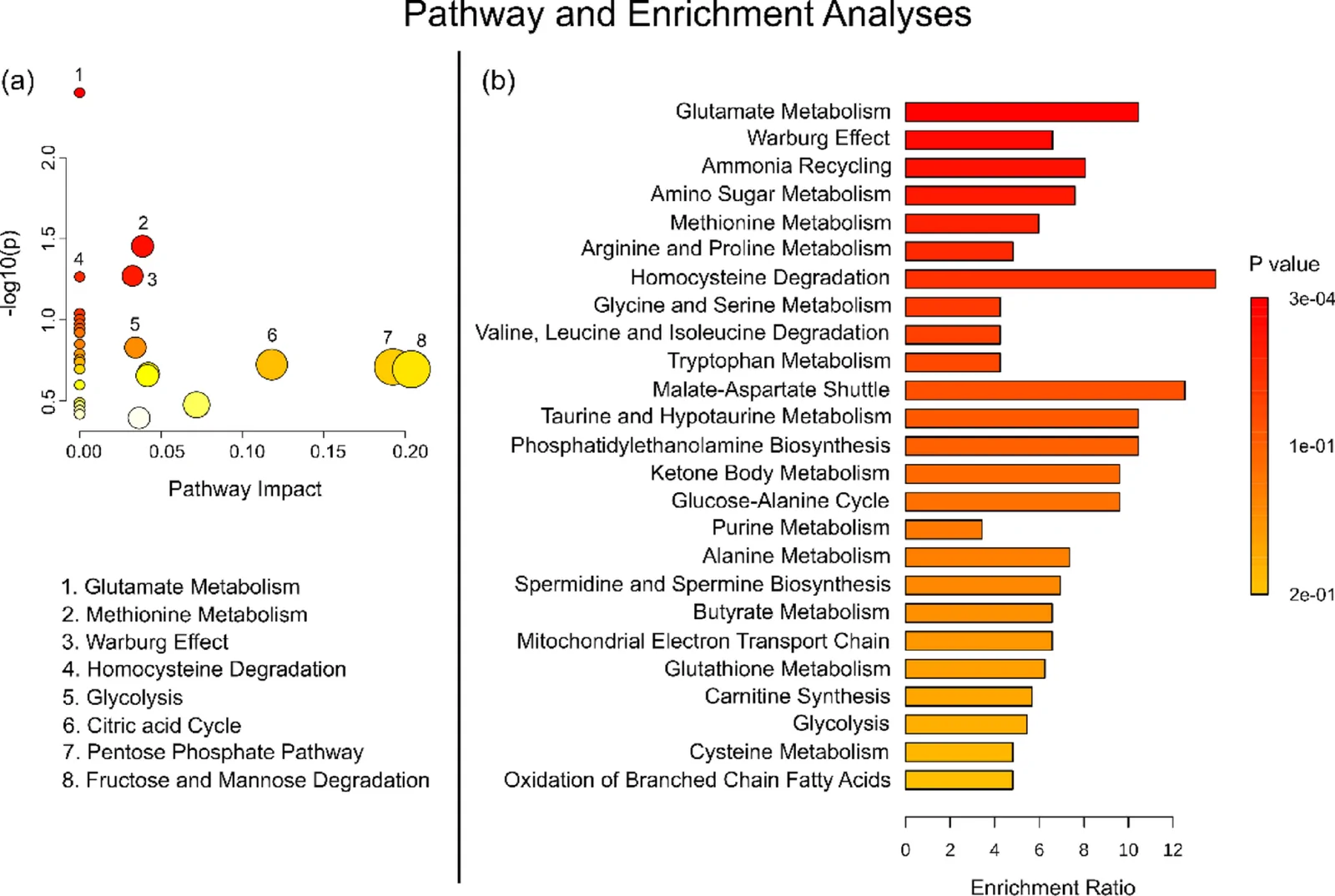
Analysis of metabolic pathways (**a**) and enrichment (**b**) for differential metabolites and lipids identified through comparing patients with CIN 2 versus CIN 3

The metabolic pathways were obtained based on the differential metabolites and lipids identified from the comparison of CIN2 versus CIN3. These include methionine metabolism, the Warburg effect, homocysteine degradation, glycolysis, the citric acid cycle, the pentose phosphate pathway, and fructose and mannose degradation (Fig. 3a). As in the previous analysis, the pathway-based enrichment analysis (Fig. 3b) confirmed that the identified metabolites are primarily involved in amino acid metabolism and degradation pathways.

Amino acid alterations are increasingly associated with the development and progression of cervical pre-cancerous cells (Khan et al. 2019; Nuer-Allornuvor et al. 2024). This may be associated with the need for a large number of amino acids to support the synthesis of proteins and nucleic acids necessary for the growth and proliferation of these cells (Jia et al. 2023; Stepka et al. 2021). Methionine, tryptophan, arginine, and proline metabolism pathways have been reported as disturbed metabolic pathways in patients with cervical cancer and its precursor lesions (Nuer-Allornuvor et al. 2024; Yin et al. 2024).

Among such pathways is glutamate metabolism, which plays a fundamental role in the biosynthesis of proteins and nucleic acids, acting in reversible or irreversible reactions catalyzed by enzymes modulated by inhibitors and activators (Yelamanchi et al. 2016). Changes in this metabolism have already been associated with several tumors, including prostate cancer (Lima et al. 2022), glioblastoma (Godlewski et al. 2025), and cervical cancer (Li and Sui 2021). In our study, the identified metabolites fructose 6-phosphate and L-glutamic acid participate in the biosynthesis of hexosamine present in this metabolism. This fact is interesting since the rate-limiting enzyme in this pathway is glutamine-fructose-6-phosphate transaminase 1 (GFPT1), which has already been reported to promote cervical cancer progression (Li et al. 2022).

Another metabolite identified in the study was guanosine-5-monophosphate (GMP), also related to the glutamate pathway. During GMP synthesis, glutamine provides amide nitrogen to xanthosine monophosphate (XMP) under the action of the enzyme GMPS and is then converted to the byproduct glutamate (Ballut et al., 2023). Studies demonstrate a positive correlation between GMPS expressivity in normal cervical tissues, cervical intraepithelial lesions, and cervical cancer tissues, which progressively increase (Wang et al., 2021). Considering that the metabolites fructose-6-phosphate, glutamic acid, and guanosine-5-monophosphate were more abundant in CIN 3 samples when compared to CIN 2 samples (Fig. 2b, d), these metabolites may reflect metabolic differences associated with lesion severity. However, we emphasize that biological conclusions about glutamate metabolism activation or lesion severity cannot be based solely on pathway enrichment results; further studies are needed to confirm these associations.

In addition, the detection of altered levels of fructose 6-phosphate and glutamic acid may reflect the energy demands of neoplastic cells. Fructose 6-phosphate is an intermediate in the glycolytic pathway; when phosphorylated by PFK-1 (phosphofructokinase-1), it generates fructose 1,6-bisphosphate, an irreversible step specific to glycolysis (Compton & Patrick, 2025; Lynch et al., 2024). Furthermore, glutamic acid, when converted to α-ketoglutarate, participates in the tricarboxylic acid (TCA) cycle in the synthesis of ATP and fatty acids (Jyotsana et al., 2022). Such metabolic changes are often associated with the “Warburg effect” (Dang, 2010), a process in which tumor cells undergo metabolic reprogramming that, instead of gaining energy from oxidative phosphorylation, prefers the absorption of glucose via aerobic glycolysis. This fact results in lactate accumulation, favoring the tumor microenvironment for the growth and proliferation of cancer (Yu et al., 2024). Specifically, in cervical cancer and its precursor lesions, there are already reports of increased lactate in CC plasma samples (de Souza Leão et al., 2025; Lyng et al., 2007). Although pathway enrichment analysis indicates involvement of processes related to glycolysis, the absence of significant variation in key metabolites in the study, such as lactate, limits the interpretation of these findings as consistent evidence of an increased glycolytic phenotype characteristic of the Warburg effect, and is not sufficient to support the occurrence of this type of metabolic reprogramming. Even so, we observed greater abundance of glutamic acid and fructose-6-phosphate in samples classified as CIN 3 compared to those of CIN 2, suggesting metabolic alterations associated with lesion progression and pointing to possible changes in cellular energy metabolism.

**Fig. 4:**
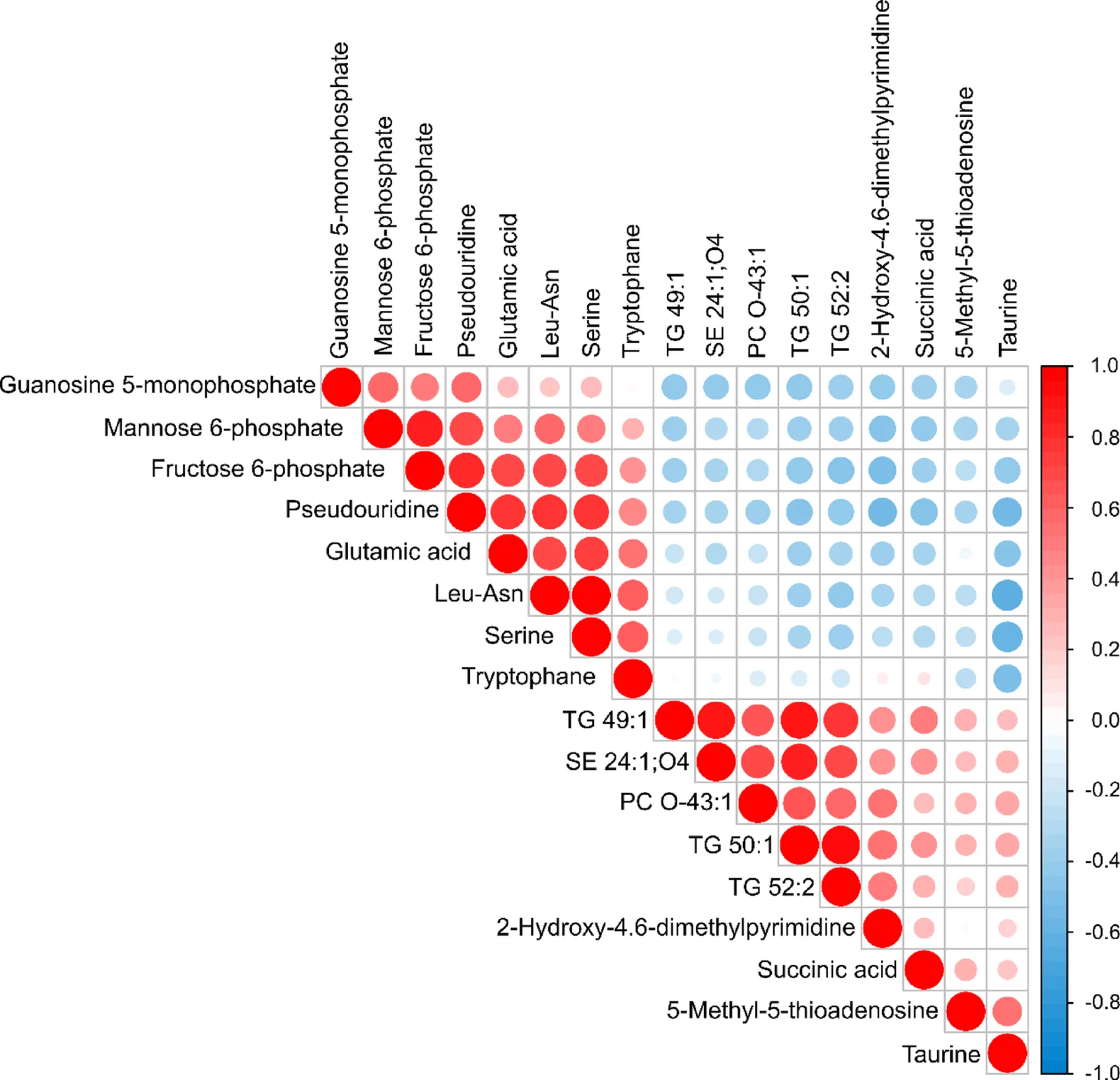
Pearson correlation matrix showing the relationship between significantly altered metabolic and lipid profiles in CIN 2 and CIN 3 samples. The circle size and color gradients of the matrix indicate the correlation coefficients, ranging from 1.0 (positive correlation) to − 1.0 (negative correlation)

After pathway and enrichment analysis, correlation analysis was conducted to investigate the relationship between the metabolic and lipid profiles of patients diagnosed with CIN 2 and CIN 3 (Fig. 4), which revealed significant correlations between these chemical compounds. As a result, it was possible to observe positive or negative correlations of metabolites and lipids among themselves, as well as four lipid subclasses that presented such correlations with metabolites that were showing differential levels in the earlier metabolomic analyses. These subclasses correspond to triglycerides (TG), oxidized phosphatidylethanolamines (PE_O), and sterol esters (SE), whose correlation levels varied according to the groups analyzed.

Considering metabolite correlations, the study revealed positive associations among tryptophan, glutamic acid, pseudouridine, and mannose-6-phosphate. Tryptophan is an essential amino acid for protein synthesis and a precursor to several biologically active compounds. In cancer progression, tryptophan is integrated into the kynurenine metabolic pathway, which is associated with the attenuation of T-cell immune responses and the inhibition of antitumor immune mechanisms (Opitz et al., 2020). Furthermore, it is known that the kynurenine pathway generates intermediate products such as 3-hydroxykynurenine (3-HK) and quinolinic acid (QA), which are associated with pro-oxidant properties (Badawy, 2022; Damerell et al., 2025; Lieu et al., 2020), and the generation of reactive oxygen species (ROS) capable of inducing damage to proteins, lipids, and DNA (Arfin et al., 2021; Ju et al., 2024). In turn, in response to oxidative stress, glutamic acid serves as a key metabolite in the synthesis of glutathione (GSH), which eliminates free radicals and other reactive oxygen species (ROS) directly and indirectly, acting as a detoxifying agent in both healthy and atypical cells (Lieu et al., 2020; Yi et al., 2020). Specifically, the presence of both metabolites has been reported at significantly elevated levels in patients with colorectal cancer (Qiu et al., 2010).

Pseudouridine is a ribonucleoside derived from uridine, that contributes to the stability, structural integrity, and translational maintenance of RNA molecules (Liu et al. 2024; Yang et al. 2025). In cancerous or proliferative tissues, studies demonstrate that under oxidative stress or nutrient deprivation, cells increase pseudouridylation of tRNAs and rRNAs, thereby ensuring the synthesis of proteins necessary for cell survival (Ding et al. 2024a, b, c; Yang et al. 2026), having been associated with neoplastic growth, metastasis and worse prognosis for patients (Ding et al. 2024a, b, c; Jia et al. 2025). Its levels have been abundantly expressed in serum and urine samples of patients with various types of cancer, such as ovarian, breast, liver, stomach, and lung cancer, and compared to healthy individuals (Liu et al. 2024a, b; Zeleznik et al. 2020). Thus, concurrent changes in the abundances of tryptophan, glutamic acid, and pseudouridine may reflect adaptive mechanisms involved in redox homeostasis and in maintaining the tumor microenvironment (Jia et al. 2025; Jin et al. 2023; Juhász et al. 2026).

In contrast, mannose-6-phosphate is a metabolite derived from mannose in the cellular environment, with its main biological functions associated with this molecule. Studies indicate that mannose is imported by the same transporter proteins as glucose and, within cells, is phosphorylated to mannose-6-phosphate (M6P). M6P is associated with the regulation of the glycolytic enzymes Hexokinase (HK) and Phosphoglucose Isomerase (PGI), which mediate glucose metabolism, thereby attenuating the Warburg effect and cell growth in tumors (Torretta et al., 2020; Zhang et al., 2022). This mechanism has been studied to improve conventional chemotherapy (Al Hadeethi et al., 2023), highlighting the potential of combined mannose- and mannose-6-phosphate-targeted delivery systems for cancer treatment (Dalle Vedove et al., 2018; Gonzalez et al., 2018).

Additionally, a positive correlation was observed between the lipids TG 50:1, TG 52:2, TG 49:1, and SE 24:1, which were significantly more abundant in CIN 2 samples when compared to CIN 3 samples (Fig. 2f). Triglycerides (TGs) are crucial in the proliferation process of cancer cells, as they provide the energy necessary for cell division processes and contribute to the maintenance of plasma membranes, thus being essential during the transformation, progression, and metastasis of cancers (Martin-Perez et al. 2022; Perrotti et al. 2016). Studies demonstrate a significant association between TG levels of dyslipidemia and the presence of CIN 2–3 (Mwangi et al. 2024) and increased risk of cervical cancer (Ashique et al. 2022; Cheng et al. 2024; Ulmer et al. 2012). TG 49:1 lipid were positively expressed in bladder cancer patients (Lee et al. 2018), TG 49:1 and TG 50:1 in lung cancer cell lines (Bin Lee et al. 2019; Yan et al. 2024), TG 50:1 and 52:2 in hepatocellular carcinoma and breast cancer (Jarc et al. 2018; Li et al. 2017).

Sterol esters (SE) are a subtype of cholesterol ester that has been linked to increased aggressiveness and progression of carcinomas (de Gonzalo-Calvo et al. 2015). To our knowledge, significant changes in SE 24:1;O4 levels were detected for the first time in this study. However, this subclass has already been associated with colorectal cancer (Liu et al. 2024a, b). Furthermore, these lipid subclasses are the main form of storage of fatty acids in the body, and they are used during hepatic homeostasis to produce ATP (Paul et al. 2022). Therefore, this behavior suggests that mechanisms of synthesis, storage, and degradation of these lipid metabolisms may be jointly regulated.

In addition, correlation analysis showed a positive interaction between the lipids TG 50:1, TG 52:2, and TG 49:1 with the metabolite’s taurine and succinic acid. Taurine contributes to regulating several cellular functions, such as osmotic regulation and calcium concentration, and modulation of the immune response against pathogens through anti-inflammatory and antioxidant effects (Lei and Mou 2020). Findings describe taurine’s ability to prevent progression of several cancers, including cervical cancer in in vitro studies (Baliou et al. 2020; Li et al. 2019). Furthermore, studies describe succinic acid as a multifunctional metabolite, capable of acting both in the immune response(Kuo et al. 2022) and in the advancement of tumor progression by regulating specific genes, such as SPP1 (osteopontin) and SLPI (leukocyte secretory protease inhibitor) in patients diagnosed with ovarian câncer (Zhao et al. 2025). In this context, studies demonstrate that reducing succinate dehydrogenase (SDH), an enzyme that catalyzes the oxidation of succinic acid in the TCA cycle, increases triglyceride levels, thereby favoring tumor metastasis. Furthermore, taurine supplementation promoted a decrease in lipogenic genes, such as SREBP, which are highly expressed in gastric, hepatocellular, colon, breast, prostate, thyroid, and glioblastoma cancers (Bao et al. 2016; Ding et al. 2024a, b, c; Prakashan et al. 2024; Li et al. 2023). Therefore, it is suggested that this positive correlation may reflect the coexistence of suppression and progression mechanisms in these neoplasms, with these lipids potentially contributing as energy reserves associated with both conditions.

Otherwise, in this study, the lipids TG 50:1, TG 52:2, and TG 49:1 were negatively correlated with serine, associated with a higher abundance of triglycerides in CIN 2 and serine in CIN 3. Serine is a fundamental precursor for the biosynthesis of proteins, essential amino acids, and nucleic acids such as pyrimidines and purines (components of cellular DNA and RNA) (Handzlik & Metallo, 2023). During carcinogenesis, this demand for serine is sustained by the *de novo* synthesis pathway (SSP), which produces it from glycolytic intermediates, and by the serine-glycine-one-carbon pathway (SGOC), which uses it as the main donor of one-carbon units for biosynthesis reactions essential to cell proliferation (Li & Ye, 2020; Su et al., 2026; Sun et al., 2023). Thus, the negative correlation between triglycerides and serine may reflect distinct metabolic demands throughout tumor progression, driven by differences in lipid storage and the synthesis of serine-derived metabolites that promote cell proliferation (Schiliro & Firestein, 2021; Tedeschi et al., 2013). This reorientation is regulated by multiple signaling pathways, including the mTOR pathway, which stimulates lipogenesis via SREBP-1 activation and interacts with the SSP pathway to regulate cell growth (Ilyassova et al., 2025; Ricoult & Manning, 2013; Szwed et al., 2021). These observations are preliminary, and functional studies will be needed to confirm the relationship between reduced lipid storage and the activation of serine metabolism during the progression of CIN.

### Differential metabolites and lipids of high-grade CINs

**Fig. 5:**
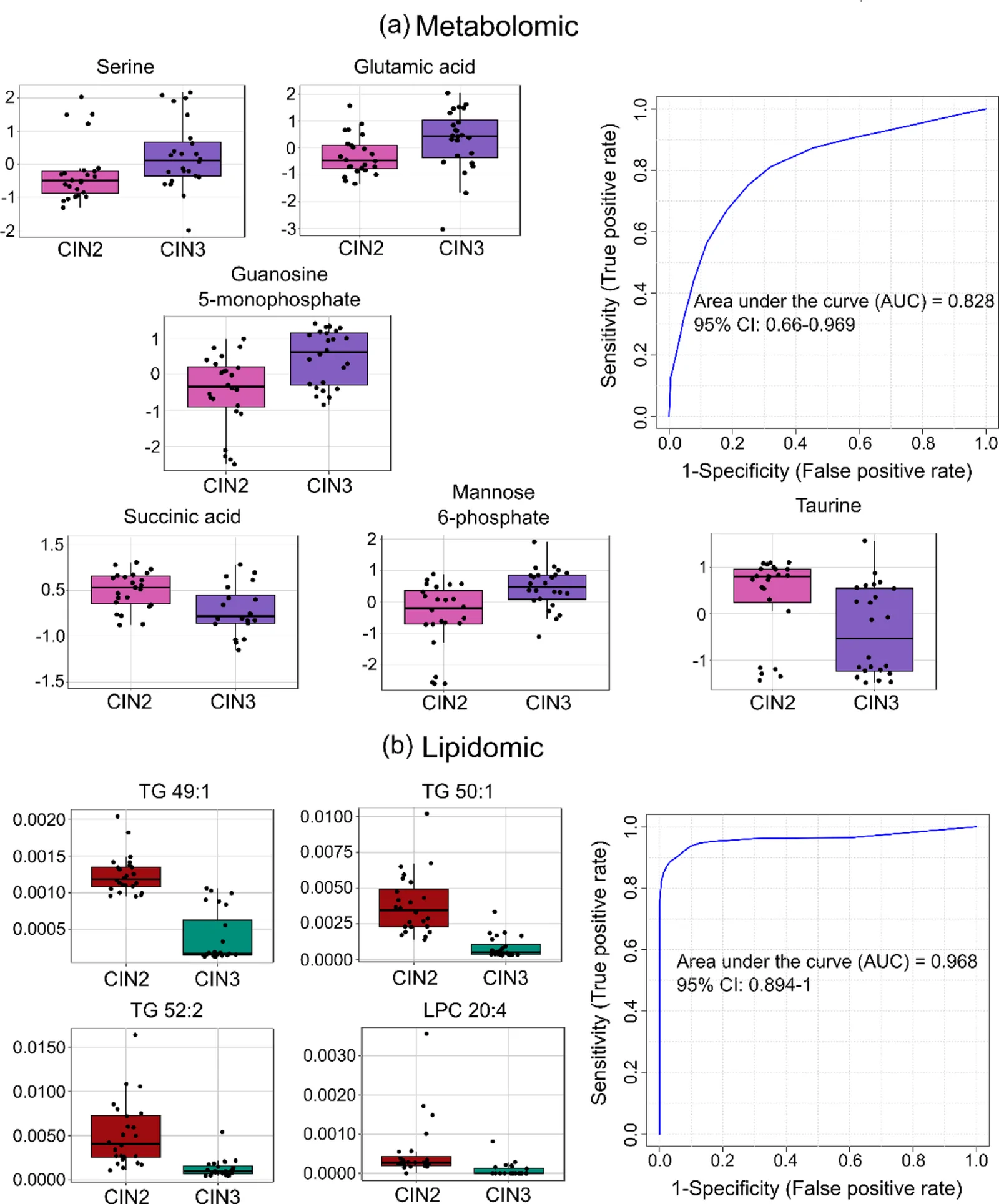
**a** Box-plot of variables related to differential metabolites with *p* < 0.001 and VIP > 1 for comparing CIN2 versus CIN3. ROC curve (upper right corner) obtained from the multivariate ROC curve analysis, demonstrating the classification efficacy for the multivariate model, with six differential metabolites as potential candidates to discriminate such lesions (AUC = 0.83, 95% CI: 0.66–0.969). **b** Box-plot of the variables related to differential lipids with *p* < 0.0001 and VIP > 1 for comparing CIN2 versus CIN3. ROC curve (right) obtained from the multivariate ROC curve analysis, demonstrating the classification efficacy for the multivariate model, with four differential lipids as potential candidates to discriminate such lesions (AUC = 0.984, 95% CI: 0.922-1.0). AUC: area under the curve; CI: confidence interval

The metabolites and lipids that were candidates for distinguish CINs were obtained from the OPLS-DA analysis (Fig. 2), and their interrelationships were described in the previous topic. ROC curve analysis evaluated these metabolites and lipids, which showed that all areas under the curve (AUC) had values above 70%. Among the 14 candidate metabolites measured, six showed a strong potential for discrimination of CIN 2 and CIN 3, exhibiting a good statistically significant difference (*p* < 0.01). These potential metabolic indicators are serine, glutamic acid, guanosine 5-monophosphate, succinic acid, mannose 6-phosphate, and taurine (Fig. 5a). Regarding lipids, among the six candidates measured, four lipids showed a strong potential for discrimination of CIN 2 and CIN 3, exhibiting an excellent statistically significant difference (*p* < 0.0001). These potential lipid indicators are TG 49:1, TG 50:1, and TG 52:2 (Fig. 5b).

Finally, as previously described, the potential metabolites serine, glutamine acid, guanonise-6-monophosphate, succinic acid, mannose 6-phosphate, and taurine are involved in essential metabolisms for protein synthesis, energy, and cell cycle maintenance, as well as modulation of the immune response in neoplasms. Since these cellular mechanisms are associated with the progression of atypical lesions, these metabolites stand out as promising candidates for composing signatures associated with NICs, and should be confirmed in future studies with independent validation. In addition, the candidate lipids TG 49:1, TG 50:1, and TG 52:2 are associated with the metabolic pathway of glycerolipids and glycerophospholipids. They are essential for mechanisms of maintenance, proliferation, and energy storage of neoplastic cells. Therefore, these lipids may play important roles in discriminating against patients with high-grade cervical lesions.

## Limitations

This exploratory pilot study has limitations that should be considered when interpreting the results. The small sample size may reduce statistical power and increase the risk of overfitting in supervised multivariate analyses. Although cross-validation and permutation testing were performed to assess model robustness, these approaches do not substitute for validation in an independent cohort; therefore, the identified discriminant metabolites and lipids should be regarded as preliminary candidates. In addition, the sample size precluded robust adjustment for potentially relevant clinical covariates, including age, BMI, menopausal status, hormone use, and smoking, and residual confounding cannot be excluded. The absence of an independent healthy cervical tissue control group also limits the biological interpretation of the molecular differences observed between CIN2 and CIN3. Larger, well-characterized cohorts with appropriate covariate adjustment and independent validation are therefore required to confirm the reproducibility and generalizability of these metabolomic and lipidomic signatures.

## Conclusion

In this study, the LC-HRMS-based metabolomic and lipidomic approach applied to cervical tissue allowed the identification of sets of metabolites and lipids differentially abundant between CIN 2 and CIN 3, indicating the presence of molecular patterns associated with the degree of lesion. Multivariate analyses revealed variables with the greatest contribution to the distinction between groups, suggesting that metabolic and lipid changes may reflect relevant biological differences in the context of high-grade cervical lesions. The metabolites serine, glutamic acid, guanonise-6-monophosphate, succinic acid, mannose 6-phosphate, and taurine and the lipids TG 49:1, TG 50:1, and TG 52:2, were potential candidates for the differentiation of CIN 2 and CIN 3. It is expected that the results obtained in this study will stimulate the use of complementary technologies in the future for more excellent monitoring of this disease, thus assisting in more individualized treatments for patients affected by precancerous lesions.

## Supplementary Information

Below is the link to the electronic supplementary material.

Supplementary Material 1.

Supplementary Material 2.

Supplementary Material 3.

## Data Availability

Fig. S1-S6 and Tables S1-S11 are provided as Supplementary Material. The raw LC-HRMS files can be found at the MassIVE Repository (Dataset MSV000103156).

## References

[CR1] Al Hadeethi, S., El-Baba, C., Araji, K., Hayar, B., Cheikh, I. A., El-Khoury, R., et al. (2023). Mannose Inhibits the Pentose Phosphate Pathway in Colorectal Cancer and Enhances Sensitivity to 5-Fluorouracil Therapy. *Cancers*, *15*(8), 2268. 10.3390/cancers15082268 PMC10137209 37190196

[CR2] Alder, S., Megyessi, D., Sundström, K., Östensson, E., Mints, M., Belkić, K., et al. (2020). Incomplete excision of cervical intraepithelial neoplasia as a predictor of the risk of recurrent disease—a 16-year follow-up study. *American Journal of Obstetrics and Gynecology*, *222*(2), 172e1–17172. 10.1016/j.ajog.2019.08.042 31473226

[CR3] Alves, M., da Silva, A. C., Torres, C., de Almeida, L., Mastella, A. M., Borges, R., & Garrett, R. (2021). A Systematic Pipeline to Enhance the Fecal Metabolome Coverage by LC-HRMS. *Journal of the Brazilian Chemical Society*. 10.21577/0103-5053.20210042

[CR4] Arfin, S., Jha, N. K., Jha, S. K., Kesari, K. K., Ruokolainen, J., Roychoudhury, S., et al. (2021). Oxidative Stress in Cancer Cell Metabolism. *Antioxidants*, *10*(5), 642. 10.3390/antiox10050642 PMC8143540 33922139

[CR5] Ashique, S., Upadhyay, A., Kumar, N., Chauhan, S., & Mishra, N. (2022). Metabolic syndromes responsible for cervical cancer and advancement of nanocarriers for efficient targeted drug delivery- A review. *Advances in Cancer Biology - Metastasis*, *4*, 100041. 10.1016/j.adcanc.2022.100041

[CR6] Athanasiou, A., Veroniki, A. A., Efthimiou, O., Kalliala, I., Naci, H., Bowden, S., et al. (2022). Comparative effectiveness and risk of preterm birth of local treatments for cervical intraepithelial neoplasia and stage IA1 cervical cancer: a systematic review and network meta-analysis. *The Lancet Oncology*, *23*(8), 1097–1108. 10.1016/S1470-2045(22)00334-5 PMC9630146 35835138

[CR7] Badawy, A. A. B. (2022). Tryptophan metabolism and disposition in cancer biology and immunotherapy. *Bioscience Reports*. 10.1042/BSR20221682 PMC9653095 36286592

[CR8] Baliou, S., Kyriakopoulos, A., Spandidos, D., & Zoumpourlis, V. (2020). Role of taurine, its haloamines and its lncRNA TUG1 in both inflammation and cancer progression. On the road to therapeutics? (Review). *International Journal of Oncology*, *57*(3), 631–664. 10.3892/ijo.2020.5100 PMC7384849 32705269

[CR9] Ballut, L., Violot, S., Kumar, S., Aghajari, N., & Balaram, H. (2023). GMP Synthetase: Allostery, Structure, and Function. *Biomolecules*, *13*(9), 1379. 10.3390/biom13091379 PMC10526850 37759779

[CR10] Bao, J., Zhu, L., Zhu, Q., Su, J., Liu, M., & Huang, W. (2016). SREBP-1 is an independent prognostic marker and promotes invasion and migration in breast cancer. *Oncology Letters*, *12*(4), 2409–2416. 10.3892/ol.2016.4988 PMC5038874 27703522

[CR11] Bergqvist, L., Virtanen, A., Kalliala, I., Bützow, R., Jakobsson, M., Heinonen, A., et al. (2025). Predictors for regression and progression of actively surveilled cervical intraepithelial neoplasia grade 2: A prospective cohort study. *Acta Obstetricia et Gynecologica Scandinavica*, *104*(4), 763–773. 10.1111/AOGS.15032;PAGE:STRING:ARTICLE/CHAPTER. 10.1111/aogs.15032 PMC11919748 39930620

[CR12] Brun, J. L., Letoffet, D., Marty, M., Griffier, R., Ah-Kit, X., & Garrigue, I. (2021). Factors predicting the spontaneous regression of cervical high-grade squamous intraepithelial lesions (HSIL/CIN2). *Archives of Gynecology and Obstetrics*, *303*(4), 1065–1073. 10.1007/s00404-020-05853-3 33175197

[CR13] Carreon, J. D., Sherman, M. E., Guillén, D., Solomon, D., Herrero, R., Jerónimo, J., et al. (2007). CIN2 is a much less reproducible and less valid diagnosis than CIN3: results from a histological review of population-based cervical samples. *International Journal of Gynecological Pathology*, *26*(4), 441–446. 10.1097/PGP.0B013E31805152AB 17885496

[CR14] Cheng, F., Wen, Z., Feng, X., Wang, X., & Chen, Y. (2020). A serum lipidomic strategy revealed potential lipid biomarkers for early-stage cervical cancer. *Life Sciences*, *260*, 118489. 10.1016/j.lfs.2020.118489 32976882

[CR15] Cheng, L., Li, Z., Zheng, Q., & Yao, Q. (2024). Correlation study of serum lipid levels and lipid metabolism-related genes in cervical cancer. *Frontiers in Oncology*. 10.3389/fonc.2024.1384778 PMC11109420 38779100

[CR16] Compton, J. A., & Patrick, W. M. (2025). The more we learn, the more diverse it gets: structures, functions and evolution in the Phosphofructokinase Superfamily. *Biochemical Journal*, *482*(09), 467–483. 10.1042/BCJ20253024 PMC12203962 40329473

[CR17] Dalle Vedove, E., Costabile, G., & Merkel, O. M. (2018). Mannose and Mannose-6‐Phosphate Receptor–Targeted Drug Delivery Systems and Their Application in Cancer Therapy. *Advanced Healthcare Materials*. 10.1002/adhm.201701398 PMC6108418 29719138

[CR18] Damerell, V., Klaassen-Dekker, N., Brezina, S., Ose, J., Ulvik, A., van Roekel, E. H., et al. (2025). Circulating tryptophan–kynurenine pathway metabolites are associated with all‐cause mortality among patients with stage I–III colorectal cancer. *International Journal of Cancer*, *156*(3), 552–565. 10.1002/ijc.35183 PMC11621991 39308420

[CR19] Dang, C. V. (2010). Rethinking the Warburg Effect with Myc Micromanaging Glutamine Metabolism. *Cancer Research*, *70*(3), 859–862. 10.1158/0008-5472.CAN-09-3556 PMC2818441 20086171

[CR20] de Gonzalo-Calvo, D., López-Vilaró, L., Nasarre, L., Perez-Olabarria, M., Vázquez, T., Escuin, D., et al. (2015). Intratumor cholesteryl ester accumulation is associated with human breast cancer proliferation and aggressive potential: a molecular and clinicopathological study. *Bmc Cancer*, *15*(1), 460. 10.1186/s12885-015-1469-5 PMC4460760 26055977

[CR27] de la Gil, A., Godzien, J., Fernández López, M., Rupérez, F. J., Barbas, C., & Otero, A. (2018). Knowledge-based metabolite annotation tool: CEU Mass Mediator. *Journal of Pharmaceutical and Biomedical Analysis*, *154*, 138–149. 10.1016/j.jpba.2018.02.046 29547800

[CR21] de Souza Leão, L. Q., de Andrade, J. C., Marques, G. M., Guimarães, C. C., de Albuquerque, V., de Silva, R., A. S., et al. (2025). Rapid prediction of cervical cancer and high-grade precursor lesions: An integrated approach using low-field 1H NMR and chemometric analysis. *Clinica Chimica Acta*, *574*, 120346. 10.1016/j.cca.2025.120346 40334834

[CR22] Del Pino, M., Angeles, M. A., Martí, C., Henere, C., Munmany, M., Marimon, L., et al. (2021). Colposcopic Impression Has a Key Role in the Estimation of the Risk of HSIL/CIN3. *Cancers 2021*, *13*(6), 1224. 10.3390/CANCERS13061224. *13*. PMC7999816 33799584

[CR25] Ding, T., Li, L., Duan, R., Chen, Y., Yang, B., & Xi, M. (2023). Risk factors analysis of recurrent disease after treatment with a loop electrosurgical excision procedure for high-grade cervical intraepithelial neoplasia. *International Journal of Gynecology & Obstetrics*, *160*(2), 538–547. 10.1002/ijgo.14340 PMC10087663 35810389

[CR23] Ding, H., Liu, N., Wang, Y., Adam, S. A., Jin, J., Feng, W., & Sun, J. (2024a). Implications of RNA pseudouridylation for cancer biology and therapeutics: a narrative review. *Journal of Translational Medicine*, *22*(1), 906. 10.1186/s12967-024-05687-6 PMC11457414 39375731

[CR24] Ding, J., Bansal, M., Cao, Y., Ye, B., Mao, R., Gupta, A., et al. (2024b). MYC Drives mRNA Pseudouridylation to Mitigate Proliferation-Induced Cellular Stress during Cancer Development. *Cancer Research*, *84*(23), 4031–4048. 10.1158/0008-5472.CAN-24-1102 PMC11609627 39240686

[CR26] Ding, Z., Wei, Y., Dai, J., Pan, C., Yang, L., Li, Q., et al. (2024c). Deficiency of SDHC promotes metastasis by reprogramming fatty acid metabolism in colorectal cancer. *Journal of Translational Medicine*, *22*(1), 544. 10.1186/s12967-024-05361-x PMC11157952 38844980

[CR28] Godlewski, A., Mojsak, P., Pienkowski, T., Lyson, T., Mariak, Z., Reszec, J., et al. (2025). Metabolomic profiling of plasma from glioma and meningioma patients based on two complementary mass spectrometry techniques. *Metabolomics*, *21*(2), 33. 10.1007/s11306-025-02231-5 39987409

[CR29] Gonzalez, P. S., O’Prey, J., Cardaci, S., Barthet, V. J. A., Sakamaki, J., Beaumatin, F., et al. (2018). Mannose impairs tumour growth and enhances chemotherapy. *Nature*, *563*(7733), 719–723. 10.1038/s41586-018-0729-3 30464341

[CR30] Guijas, C., Montenegro-Burke, J. R., Domingo-Almenara, X., Palermo, A., Warth, B., Hermann, G., et al. (2018). METLIN: A Technology Platform for Identifying Knowns and Unknowns. *Analytical Chemistry*, *90*(5), 3156–3164. 10.1021/acs.analchem.7b04424 PMC5933435 29381867

[CR31] Handzlik, M. K., & Metallo, C. M. (2023). Sources and Sinks of Serine in Nutrition, Health, and Disease. *Annual Review of Nutrition*, *43*(1), 123–151. 10.1146/annurev-nutr-061021-022648 PMC10783795 37307855

[CR32] Ilyassova, B., Rakhimgerey, N., Rakhimova, S., Satvaldina, N., Daniyarov, A., Akilzhanova, A., et al. (2025). The Adaptation of Cancer Cells to Serum Deprivation Is Mediated by mTOR-Dependent Cholesterol Synthesis. *International Journal of Molecular Sciences*, *26*(22), 10932. 10.3390/ijms262210932 PMC12652518 41303417

[CR33] International Agency for Research on Cancer (2025). January 22). *Cancer Today*. https://gco.iarc.who.int/today/

[CR34] Jarc, E., Eichmann, T. O., Zimmermann, R., & Petan, T. (2018). Lipidomic data on lipid droplet triglyceride remodelling associated with protection of breast cancer cells from lipotoxic stress. *Data in Brief*, *18*, 234–240. 10.1016/j.dib.2018.03.033 PMC5996238 29896513

[CR36] Jia, Y., Zou, K., & Zou, L. (2023). Research progress of metabolomics in cervical cancer. *European Journal of Medical Research*, *28*(1), 586. 10.1186/s40001-023-01490-z PMC10717910 38093395

[CR35] Jia, S., Yu, X., Deng, N., Zheng, C., Ju, M., Wang, F., et al. (2025). Deciphering the pseudouridine nucleobase modification in human diseases: From molecular mechanisms to clinical perspectives. *Clinical and Translational Medicine*. 10.1002/ctm2.70190 PMC11746961 39834094

[CR37] Jin, J., Byun, J. K., Choi, Y. K., & Park, K. G. (2023). Targeting glutamine metabolism as a therapeutic strategy for cancer. *Experimental & Molecular Medicine*, *55*(4), 706–715. 10.1038/s12276-023-00971-9 PMC10167356 37009798

[CR38] Ju, S., Singh, M. K., Han, S., Ranbhise, J., Ha, J., Choe, W., et al. (2024). Oxidative Stress and Cancer Therapy: Controlling Cancer Cells Using Reactive Oxygen Species. *International Journal of Molecular Sciences*, *25*(22), 12387. 10.3390/ijms252212387 PMC11595237 39596452

[CR39] Juhász, L., Galla, Z., Tanaka, M., & Vécsei, L. (2026). Receptor–Mitochondria Crosstalk in the Kynurenine Metabolic Pathway: Integrating Metabolomics and Clinical Mass Spectrometry. *Antioxidants*, *15*(2), 261. 10.3390/antiox15020261 PMC12938270 41750641

[CR40] Juliato, C. R. T., Teixeira, J. C., Derchain, S. F. M., Barbosa, S. B., Martinez, E. Z., Panetta, K., & de Angelo-Andrade, L. A. (2000). Correlação entre o Diagnóstico Histológico da Biópsia e o da Conização por Cirurgia de Alta Freqüência por Alça (CAF) no Tratamento da Neoplasia Intra-epitelial Cervical. *Revista Brasileira de Ginecologia e Obstetrícia*, *22*(2), 65–70. 10.1590/S0100-72032000000200002

[CR41] Jyotsana, N., Ta, K. T., & DelGiorno, K. E. (2022). The Role of Cystine/Glutamate Antiporter SLC7A11/xCT in the Pathophysiology of Cancer. *Frontiers in Oncology*. 10.3389/fonc.2022.858462 PMC8904967 35280777

[CR42] Karikkakkavil Prakashan, A. D., Muthukumar, S. P., & Martin, A. (2024). Taurine Activates SIRT1/AMPK/FOXO1 Signaling Pathways to Favorably Regulate Lipid Metabolism in C57BL6 Obese Mice. *Molecular Nutrition & Food Research*. 10.1002/mnfr.202200660 38549461

[CR43] Khan, I., Nam, M., Kwon, M., Seo, S. S., Jung, S., Han, J. S., et al. (2019). LC/MS-Based Polar Metabolite Profiling Identified Unique Biomarker Signatures for Cervical Cancer and Cervical Intraepithelial Neoplasia Using Global and Targeted Metabolomics. *Cancers 2019*, *11*(4), 511. 10.3390/CANCERS11040511. *11*. PMC6521312 30974861

[CR44] Kuo, C. C., Wu, J. Y., & Wu, K. K. (2022). Cancer-derived extracellular succinate: a driver of cancer metastasis. *Journal of Biomedical Science*, *29*(1), 93. 10.1186/s12929-022-00878-z PMC9641777 36344992

[CR46] Lee, M. Y., Yeon, A., Shahid, M., Cho, E., Sairam, V., Figlin, R., et al. (2018). Reprogrammed lipid metabolism in bladder cancer with cisplatin resistance. *Oncotarget*, *9*(17), 13231–13243. 10.18632/oncotarget.24229 PMC5862574 29568353

[CR45] Lee, G., Bin, Lee, J. C., & Moon, M. H. (2019). Plasma lipid profile comparison of five different cancers by nanoflow ultrahigh performance liquid chromatography-tandem mass spectrometry. *Analytica Chimica Acta*, *1063*, 117–126. 10.1016/j.aca.2019.02.021 30967175

[CR47] Lei, L., & Mou, Q. (2020). Exosomal taurine up-regulated 1 promotes angiogenesis and endothelial cell proliferation in cervical cancer. *Cancer Biology & Therapy*, *21*(8), 717–725. 10.1080/15384047.2020.1764318 PMC7515536 32432954

[CR49] Li, B., & Sui, L. (2021). Metabolic reprogramming in cervical cancer and metabolomics perspectives. *Nutrition & Metabolism*, *18*(1), 93. 10.1186/s12986-021-00615-7 PMC8525007 34666780

[CR48] Li, A. M., & Ye, J. (2020). Reprogramming of serine, glycine and one-carbon metabolism in cancer. *Biochimica et Biophysica Acta (BBA) - Molecular Basis of Disease*, *1866*(10), 165841. 10.1016/j.bbadis.2020.165841 PMC7442608 32439610

[CR53] Li, Z., Guan, M., Lin, Y., Cui, X., Zhang, Y., Zhao, Z., & Zhu, J. (2017). Aberrant Lipid Metabolism in Hepatocellular Carcinoma Revealed by Liver Lipidomics. *International Journal of Molecular Sciences*, *18*(12), 2550. 10.3390/ijms18122550 PMC5751153 29182572

[CR51] Li, H., Ruan, W. J., Liu, L. Q., Wan, H. F., Yang, X. H., Zhu, W. F., et al. (2019). Impact of Taurine on the proliferation and apoptosis of human cervical carcinoma cells and its mechanism. *Chinese Medical Journal*, *132*(8), 948–956. 10.1097/CM9.0000000000000162 PMC6595772 30958437

[CR50] Li, D., Guan, M., Cao, X., Zha, Z. Q., Zhang, P., Xiang, H., et al. (2022). GFPT1 promotes the proliferation of cervical cancer via regulating the ubiquitination and degradation of PTEN. *Carcinogenesis*, *43*(10), 969–979. 10.1093/carcin/bgac073 36040914

[CR52] Li, Y., Wu, S., Zhao, X., Hao, S., Li, F., Wang, Y., et al. (2023). Key events in cancer: Dysregulation of SREBPs. *Frontiers in Pharmacology*. 10.3389/fphar.2023.1130747 PMC10030587 36969840

[CR54] Liebisch, G., Fahy, E., Aoki, J., Dennis, E. A., Durand, T., Ejsing, C. S., et al. (2020). Update on LIPID MAPS classification, nomenclature, and shorthand notation for MS-derived lipid structures. *Journal of Lipid Research*, *61*(12), 1539–1555. 10.1194/jlr.S120001025 PMC7707175 33037133

[CR55] Lieu, E. L., Nguyen, T., Rhyne, S., & Kim, J. (2020). Amino acids in cancer. *Experimental & Molecular Medicine*, *52*(1), 15–30. 10.1038/s12276-020-0375-3 PMC7000687 31980738

[CR56] Lima, A. R., Carvalho, M., Aveiro, S. S., Melo, T., Domingues, M. R., Macedo-Silva, C., et al. (2022). Comprehensive Metabolomics and Lipidomics Profiling of Prostate Cancer Tissue Reveals Metabolic Dysregulations Associated with Disease Development. *Journal of Proteome Research*, *21*(3), 727–739. 10.1021/acs.jproteome.1c00754 34813334

[CR57] Liu, C., Xie, J., Ye, B., Zhong, J., & Xu, X. (2024a). Unraveling the link between sterol ester and colorectal cancer: a two-sample mendelian randomization study. *Bmc Cancer*, *24*(1), 1462. 10.1186/s12885-024-13228-z PMC11603857 39604873

[CR58] Liu, K., Zhang, S., Liu, Y., Hu, X., & Gu, X. (2024b). Advancements in pseudouridine modifying enzyme and cancer. *Frontiers in Cell and Developmental Biology*. 10.3389/fcell.2024.1465546 PMC11683142 39737343

[CR59] Liu W. W., Zheng, S. Q., Li, T., Fei, Y. F., Wang, C., Zhang, S., et al. (2024). RNA modifications in cellular metabolism: implications for metabolism-targeted therapy and immunotherapy. *Signal Transduction and Targeted Therapy*, *9*(1), 70. 10.1038/s41392-024-01777-5 PMC10966055 38531882

[CR60] Loopik, D. L., Bentley, H. A., Eijgenraam, M. N., IntHout, J., Bekkers, R. L. M., & Bentley, J. R. (2021). The Natural History of Cervical Intraepithelial Neoplasia Grades 1, 2, and 3: A Systematic Review and Meta-analysis. *Journal of Lower Genital Tract Disease*, *25*(3), 221–231. 10.1097/LGT.0000000000000604 34176914

[CR61] Lycke, K. D., Kahlert, J., Damgaard, R. K., Eriksen, D. O., Bennetsen, M. H., Gravitt, P. E., et al. (2023). Clinical course of cervical intraepithelial neoplasia grade 2: a population-based cohort study. *American Journal of Obstetrics and Gynecology*, *229*(6), 656e1–65656. 10.1016/j.ajog.2023.08.008 37595822

[CR62] Lynch, E. M., Hansen, H., Salay, L., Cooper, M., Timr, S., Kollman, J. M., & Webb, B. A. (2024). Structural basis for allosteric regulation of human phosphofructokinase-1. *Nature Communications*, *15*(1), 7323. 10.1038/s41467-024-51808-6 PMC11345425 39183237

[CR63] Lyng, H., Sitter, B., Bathen, T. F., Jensen, L. R., Sundfør, K., Kristensen, G. B., & Gribbestad, I. S. (2007). Metabolic mapping by use of high-resolution magic angle spinning 1H MR spectroscopy for assessment of apoptosis in cervical carcinomas. *Bmc Cancer*, *7*(1), 11. 10.1186/1471-2407-7-11 PMC1781459 17233882

[CR64] Macdonald, M., Smith, J. H. F., Tidy, J. A., & Palmer, J. E. (2018). Conservative management of CIN2: National Audit of British Society for Colposcopy and Cervical Pathology members’ opinion. *Journal of Obstetrics and Gynaecology*, *38*(3), 388–394. 10.1080/01443615.2017.1316973 29212399

[CR65] Martin-Perez, M., Urdiroz-Urricelqui, U., Bigas, C., & Benitah, S. A. (2022). The role of lipids in cancer progression and metastasis. *Cell Metabolism*, *34*(11), 1675–1699. 10.1016/j.cmet.2022.09.023 36261043

[CR66] Matyash, V., Liebisch, G., Kurzchalia, T. V., Shevchenko, A., & Schwudke, D. (2008). Lipid extraction by methyl-tert-butyl ether for high-throughput lipidomics. *Journal of Lipid Research*, *49*(5), 1137–1146. 10.1194/jlr.D700041-JLR200 PMC2311442 18281723

[CR67] Mwangi, G. F., Niyonzima, N., Atwine, R., Tusubira, D., Mugyenyi, G. R., & Ssedyabane, F. (2024). Dyslipidemia: prevalence and association with precancerous and cancerous lesions of the cervix; a pilot study. *Lipids in Health and Disease*, *23*(1), 3. 10.1186/s12944-023-01997-8 PMC10770978 38184564

[CR68] Nam, M., Seo, S. S., Jung, S., Jang, S. Y., Lee, J., Kwon, M., et al. (2021). Comparable Plasma Lipid Changes in Patients with High-Grade Cervical Intraepithelial Neoplasia and Patients with Cervical Cancer. *Journal of Proteome Research*, *20*(1), 740–750. 10.1021/acs.jproteome.0c00640 33241689

[CR69] Nuer-Allornuvor, G. F., Alolga, R. N., Liang, S., Ling, Z., Jingjing, W., Xu, B. Q., et al. (2024). GC–MS-based untargeted plasma metabolomics identifies a 2-biomarker panel for possible diagnosis of precancerous cervical intraepithelial neoplasia stages from cervical cancer. *Scientific Reports*, *14*(1), 17649. 10.1038/s41598-024-64574-8 PMC11291879 39085248

[CR70] Opitz, C. A., Patterson, S., Mohapatra, L. F., Dewi, S. R., Sadik, D. L., Platten, A., M., & Trump, S. (2020). The therapeutic potential of targeting tryptophan catabolism in cancer. *British Journal of Cancer*, *122*(1), 30–44. 10.1038/s41416-019-0664-6 PMC6964670 31819194

[CR71] Östör, A. G. M. D. (1993). Natural History of Cervical Intraepithelial Neoplasia A Critical Review. *International Journal of Gynecological Pathology*, *12*(2), 186–192. 8463044

[CR72] Paul, B., Lewinska, M., & Andersen, J. B. (2022). Lipid alterations in chronic liver disease and liver cancer. *JHEP Reports*, *4*(6), 100479. 10.1016/j.jhepr.2022.100479 PMC9034302 35469167

[CR73] Perrotti, F., Rosa, C., Cicalini, I., Sacchetta, P., Del Boccio, P., Genovesi, D., & Pieragostino, D. (2016). Advances in Lipidomics for Cancer Biomarkers Discovery. *International Journal of Molecular Sciences*, *17*(12), 1992. 10.3390/ijms17121992 PMC5187792 27916803

[CR74] Qiu, Y., Cai, G., Su, M., Chen, T., Liu, Y., Xu, Y., et al. (2010). Urinary Metabonomic Study on Colorectal Cancer. *Journal of Proteome Research*, *9*(3), 1627–1634. 10.1021/pr901081y 20121166

[CR75] Ricoult, S. J. H., & Manning, B. D. (2013). The multifaceted role of mTORC1 in the control of lipid metabolism. *The EMBO Reports*, *14*(3), 242–251. 10.1038/embor.2013.5 PMC3589096 23399656

[CR76] Schiliro, C., & Firestein, B. L. (2021). Mechanisms of Metabolic Reprogramming in Cancer Cells Supporting Enhanced Growth and Proliferation. *Cells*, *10*(5), 1056. 10.3390/cells10051056 PMC8146072 33946927

[CR77] Silva, A. C. R., da Silva, C. C., Garrett, R., & Rezende, C. M. (2020). Comprehensive lipid analysis of green Arabica coffee beans by LC-HRMS/MS. *Food Research International*, *137*, 109727. 10.1016/j.foodres.2020.109727 33233296

[CR78] Silva, A. C. R., Garrett, R., Rezende, C. M., & Meckelmann, S. W. (2022). Lipid characterization of arabica and robusta coffee beans by liquid chromatography-ion mobility-mass spectrometry. *Journal of Food Composition and Analysis*, *111*, 104587. 10.1016/j.jfca.2022.104587

[CR79] Starodubtseva, N. L., Chagovets, V. V., Nekrasova, M. E., Nazarova, N. M., Tokareva, A. O., Bourmenskaya, O. V., et al. (2022). Shotgun Lipidomics for Differential Diagnosis of HPV-Associated Cervix Transformation. *Metabolites*, *12*(6), 503. 10.3390/METABO12060503/S1 PMC9229224 35736434

[CR80] Stepka, P., Vsiansky, V., Raudenska, M., Gumulec, J., Adam, V., & Masarik, M. (2021). Metabolic and Amino Acid Alterations of the Tumor Microenvironment. *Current Medicinal Chemistry*, *28*(7), 1270–1289. 10.2174/0929867327666200207114658 32031065

[CR81] Su, P., Yang, Y., & Zheng, H. (2026). The serine code of metabolic reprogramming: multidimensional roles of the serine synthesis pathway in tumors and novel breakthroughs for targeted therapy. *Frontiers in Immunology*. 10.3389/fimmu.2026.1779543 PMC12971912 41816347

[CR82] Sumner, L. W., Amberg, A., Barrett, D., Beale, M. H., Beger, R., Daykin, C. A., et al. (2007). Proposed minimum reporting standards for chemical analysis. *Metabolomics*, *3*(3), 211–221. 10.1007/s11306-007-0082-2 PMC3772505 24039616

[CR83] Sun, W., Liu, R., Gao, X., Lin, Z., Tang, H., Cui, H., & Zhao, E. (2023). Targeting serine-glycine-one-carbon metabolism as a vulnerability in cancers. *Biomarker Research*, *11*(1), 48. 10.1186/s40364-023-00487-4 PMC10161514 37147729

[CR84] Szwed, A., Kim, E., & Jacinto, E. (2021). Regulation and metabolic functions of mTORC1 and mTORC2. *Physiological Reviews*, *101*(3), 1371–1426. 10.1152/physrev.00026.2020 PMC8424549 33599151

[CR85] Takeda, H., Matsuzawa, Y., Takeuchi, M., Takahashi, M., Nishida, K., Harayama, T., et al. (2024). MS-DIAL 5 multimodal mass spectrometry data mining unveils lipidome complexities. *Nature Communications*, *15*(1), 9903. 10.1038/s41467-024-54137-w PMC11605090 39609386

[CR86] Tedeschi, P. M., Markert, E. K., Gounder, M., Lin, H., Dvorzhinski, D., Dolfi, S. C., et al. (2013). Contribution of serine, folate and glycine metabolism to the ATP, NADPH and purine requirements of cancer cells. *Cell Death & Disease*, *4*(10), e877–e877. 10.1038/cddis.2013.393 PMC3920946 24157871

[CR87] Tokareva, A., Chagovets, V., Attoeva, D., Starodubtseva, N., Nazarova, N., Gusakov, K., et al. (2022). Non-Invasive Differential Diagnosis of Cervical Neoplastic Lesions by the Lipid Profile Analysis of Cervical Scrapings. *Metabolites*, *12*(9), 883. 10.3390/METABO12090883/S1 PMC9506087 36144287

[CR88] Torretta, S., Scagliola, A., Ricci, L., Mainini, F., Di Marco, S., Cuccovillo, I., et al. (2020). D-mannose suppresses macrophage IL-1β production. *Nature Communications*, *11*(1), 6343. 10.1038/s41467-020-20164-6 PMC7733482 33311467

[CR89] Tsugawa, H., Cajka, T., Kind, T., Ma, Y., Higgins, B., Ikeda, K., et al. (2015). MS-DIAL: data-independent MS/MS deconvolution for comprehensive metabolome analysis. *Nature Methods*, *12*(6), 523–526. 10.1038/nmeth.3393 PMC4449330 25938372

[CR90] Ulmer, H., Bjørge, T., Concin, H., Lukanova, A., Manjer, J., Hallmans, G., et al. (2012). Metabolic risk factors and cervical cancer in the metabolic syndrome and cancer project (Me–Can). *Gynecologic Oncology*, *125*(2), 330–335. 10.1016/j.ygyno.2012.01.052 22330614

[CR91] Wang, J., Wu, Y., Li, Y., Wang, Y., Shen, F., Zhou, J., & Chen, Y. (2021). Guanosine monophosphate synthase upregulation mediates cervical cancer progression by inhibiting the apoptosis of cervical cancer cells via the Stat3/P53 pathway. *International Journal of Oncology*, *58*(4), 3. 10.3892/ijo.2021.5183 PMC7891820 33649833

[CR92] WHO. (2024). *WHO guideline for screening and treatment of cervical pre-cancer lesions for cervical cancer prevention: use of dual-stain cytology to triage women after a positive test for human papillomavirus (HPV)*. World Health Organization. 38976622

[CR93] Wishart, D. S., Feunang, Y. D., Marcu, A., Guo, A. C., Liang, K., Vázquez-Fresno, R., et al. (2018). HMDB 4.0: the human metabolome database for 2018. *Nucleic Acids Research*, *46*(D1), D608–D617. 10.1093/nar/gkx1089 PMC5753273 29140435

[CR94] Wright, T. C., Massad, L. S., Dunton, C. J., Spitzer, M., Wilkinson, E. J., & Solomon, D. (2007). 2006 consensus guidelines for the management of women with abnormal cervical cancer screening tests. *American Journal of Obstetrics and Gynecology*, *197*(4), 346–355. 10.1016/j.ajog.2007.07.047 17904957

[CR95] Yan, F., Liu, C., Song, D., Zeng, Y., Zhan, Y., Zhuang, X., et al. (2024). Integration of clinical phenoms and metabolomics facilitates precision medicine for lung cancer. *Cell Biology and Toxicology*, *40*(1), 25. 10.1007/s10565-024-09861-w PMC11063108 38691184

[CR97] Yang, H. P., Zuna, R. E., Schiffman, M., Walker, J. L., Sherman, M. E., Landrum, L. M., et al. (2012). Clinical and pathological heterogeneity of cervical intraepithelial neoplasia grade 3. *Plos One*. 10.1371/JOURNAL.PONE.0029051 PMC3258246 22253702

[CR98] Yang, Q., Al-Hendy, A., & Boyer, T. G. (2026). Molecular Insights into Widespread Pseudouridine RNA Modifications: Implications for Women’s Health and Disease. *Biology*, *15*(2), 142. 10.3390/biology15020142 PMC12837296 41594877

[CR96] Yang, Boyer, T. G., & Al-Hendy, A. (2025). Pseudouridine Synthase 7 in Cancer: Functions, Mechanisms, and Therapeutic Potential. *Cells*, *14*(17), 1380. 10.3390/cells14171380 PMC12428485 40940790

[CR99] Yelamanchi, S. D., Jayaram, S., Thomas, J. K., Gundimeda, S., Khan, A. A., Singhal, A., et al. (2016). A pathway map of glutamate metabolism. *Journal of Cell Communication and Signaling*, *10*(1), 69–75. 10.1007/s12079-015-0315-5 PMC4850134 26635200

[CR100] Yi, H., Talmon, G., & Wang, J. (2020). Glutamate in cancers: from metabolism to signaling. *The Journal of Biomedical Research*, *34*(4), 260. 10.7555/JBR.34.20190037 PMC7386414 32594024

[CR101] Yin, Z., Hua, X., & Lu, M. (2024). Integrated Network Pharmacology and Metabolomics to Dissect the Mechanisms of Naringin for Treating Cervical Cancer. *Combinatorial Chemistry & High Throughput Screening*, *27*(5), 754–764. 10.2174/1386207326666230504124030 37143280

[CR102] Yu, Y., Jiang, Y., Glandorff, C., & Sun, M. (2024). Exploring the mystery of tumor metabolism: Warburg effect and mitochondrial metabolism fighting side by side. *Cellular Signalling*, *120*, 111239. 10.1016/j.cellsig.2024.111239 38815642

[CR103] Zeleznik, O. A., Eliassen, A. H., Kraft, P., Poole, E. M., Rosner, B. A., Jeanfavre, S., et al. (2020). A Prospective Analysis of Circulating Plasma Metabolites Associated with Ovarian Cancer Risk. *Cancer Research*, *80*(6), 1357–1367. 10.1158/0008-5472.CAN-19-2567 PMC7073287 31969373

[CR104] Zhang, R., Yang, Y., Dong, W., Lin, M., He, J., Zhang, X., et al. (2022). D-mannose facilitates immunotherapy and radiotherapy of triple-negative breast cancer via degradation of PD-L1. *Proceedings of the National Academy of Sciences*. 10.1073/pnas.2114851119 PMC8872783 35181605

[CR105] Zhao, J., Guo, P., Zhao, L., & Wang, X. (2025). Exploring the mechanism of action of succinic acid in ovarian cancer via single-cell sequencing of the tumor immune microenvironment. *Frontiers in Oncology*. 10.3389/fonc.2025.1535504 PMC11973073 40196737

