# Supplementary material for: Metabolomics and lipidomics in the differentiation of CIN 2 and CIN 3: an exploratory pilot study
Source: Metabolomics. 2026 Nov 13;22(6):175. doi: 10.1007/s11306-026-02555-w (PMC13768218; doi:10.1007/s11306-026-02555-w)
Supplement: Supplementary file 1 [file 11306_2026_2555_MOESM2_ESM.pdf]

SUPPLEMENTARY MATERIAL 2

Metabolomics and lipidomics in the differentiation of CIN 2 and CIN 3: an exploratory pilot study

Luana Quadros de Souza Leão[a,b], Giovanna Melo Marques[b], Adriano de Souza Carolino[c], Ana Carolina Rosa da Silva[d,e], Sofia Angiole Cavalcante[b], Elzalina Ribeiro Soares[f], Jonas Balan de Padua[g], Anderson Ferreira Gonçalves[g], Marina Amaral Alves [d,h], Rafael Garrett[d], Priscila Ferreira de Aquino[b*]

[a] Postgraduate Program in Chemistry (PPGQ), Federal University of Amazonas (UFAM), Manaus, AM, Brazil.

[b] Leonidas and Maria Deane Institute/Fiocruz Amazônia (ILMD/Fiocruz Amazônia), Manaus, AM, Brazil.

[c] Postgraduate Program in Materials Science and Engineering (PPGCEM), Federal University of Amazonas (UFAM), Manaus, AM, Brazil.

[e] SPARC BioCentre, The Hospital for Sick Children, Toronto, ON, Canada.

[f] Department of Chemistry, Amazonas State University (UEA), Tefé, AM, Brazil.

[g] Amazonas State Oncology Control Center Foundation (FCECON), Manaus, AM, Brazil.

[h] Laboratory of Metabolomics Applied to Systems Medicine (Meta2MS), Federal University of Rio de Janeiro (IPPN/UFRJ), University City, Rio de Janeiro, RJ, Brazil.

***Corresponding author:** P. F. Aquino. E-mail: priscila.aquino@fiocruz.br, ORCID: https://orcid.org/0000-0002-2344-0493, Leonidas and Maria Deane Institute/Fiocruz Amazônia (ILMD/Fiocruz Amazônia), Manaus, AM, Brazil.

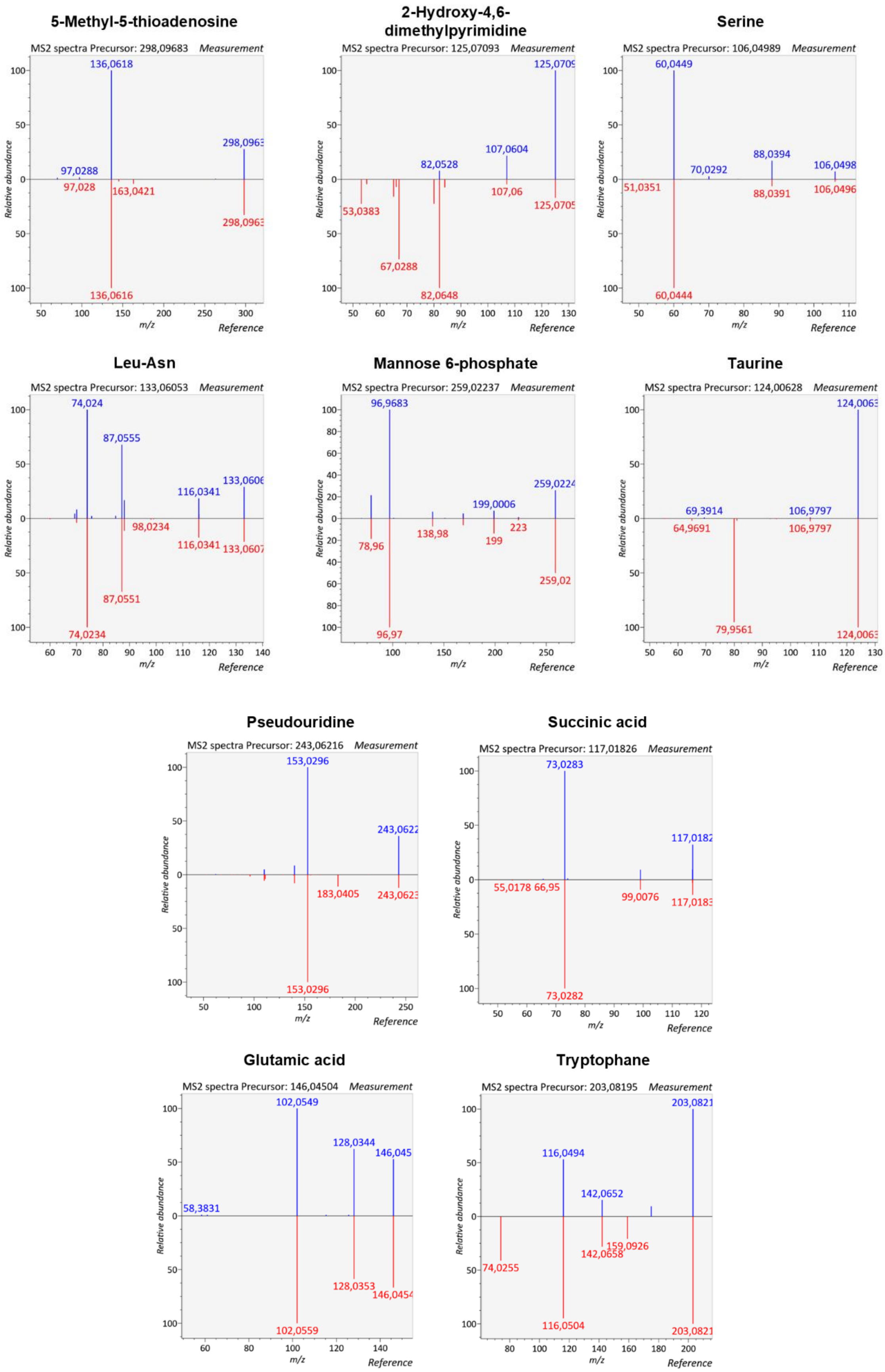

Figure S1 – Identification of metabolites from the metabolomic study (Part 1). In blue, the experimental MS2 spectrum. In red, the MS2 spectrum from the library database contained in the MS-DIAL.

- **Guanosine monophosphate**

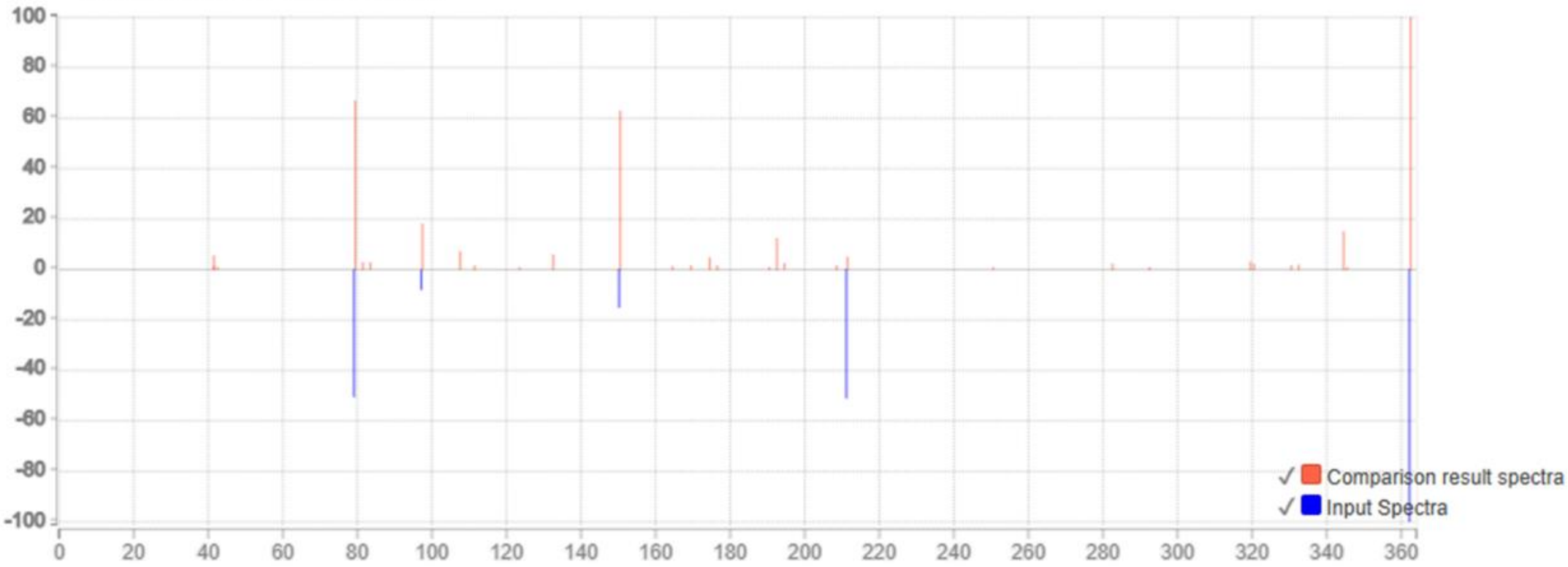

- **Fructose-6-phosphate**

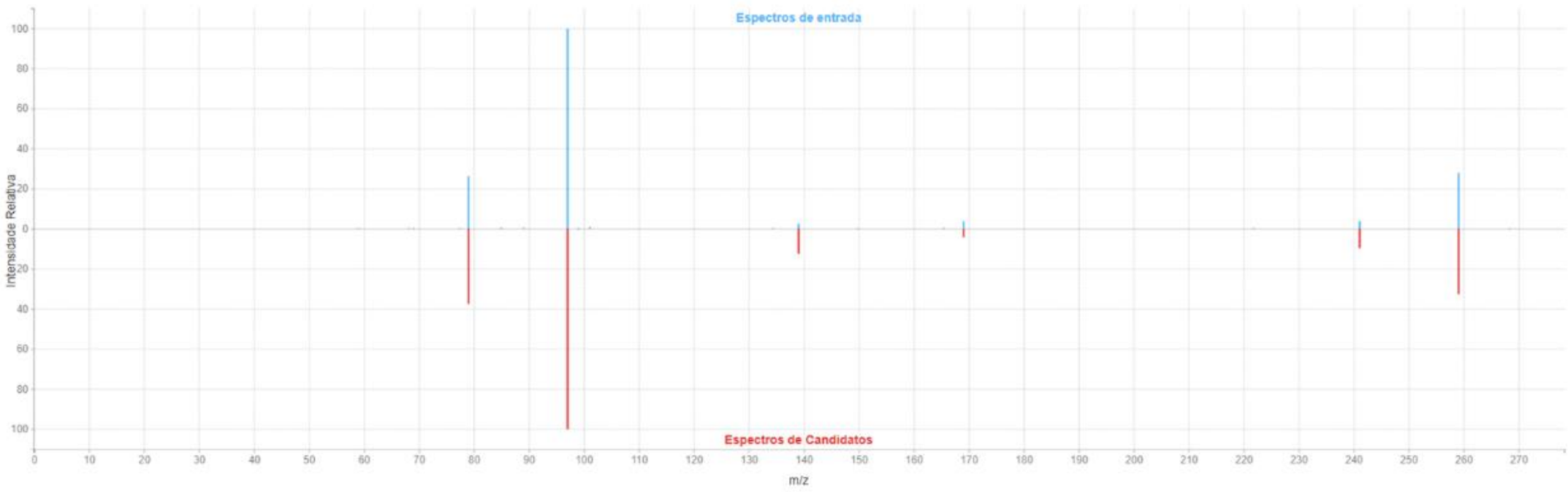

Figure S2 – Identification of metabolites from the metabolomic study (Part 2) using the CEU Mediator and CFM-ID 4.0 platforms, respectively.

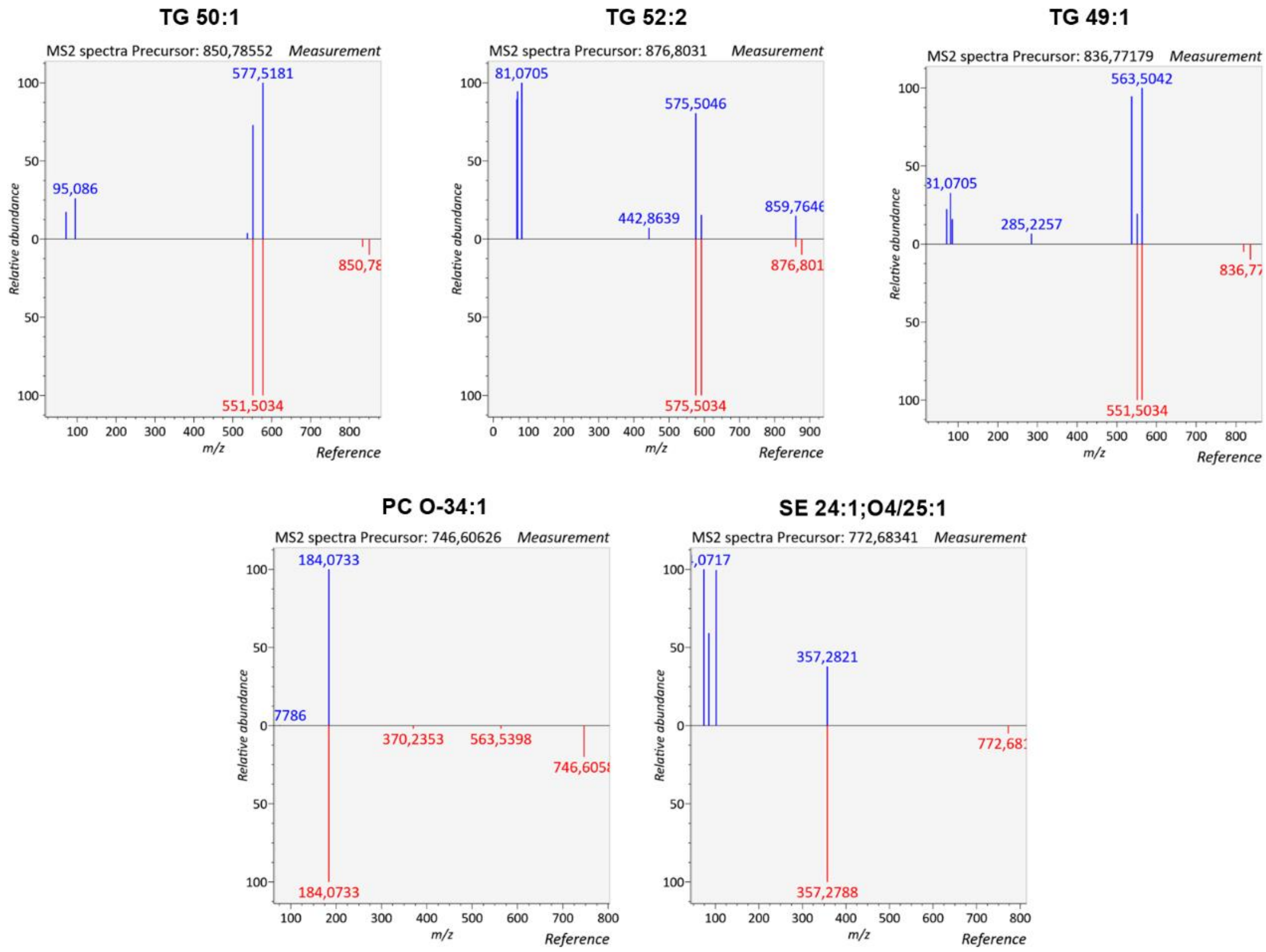

Figure S3 – Identification of lipids from the lipidomic study. In blue, the experimental MS2 spectrum. In red, the MS2 spectrum from the library database contained in the MS-DIAL.

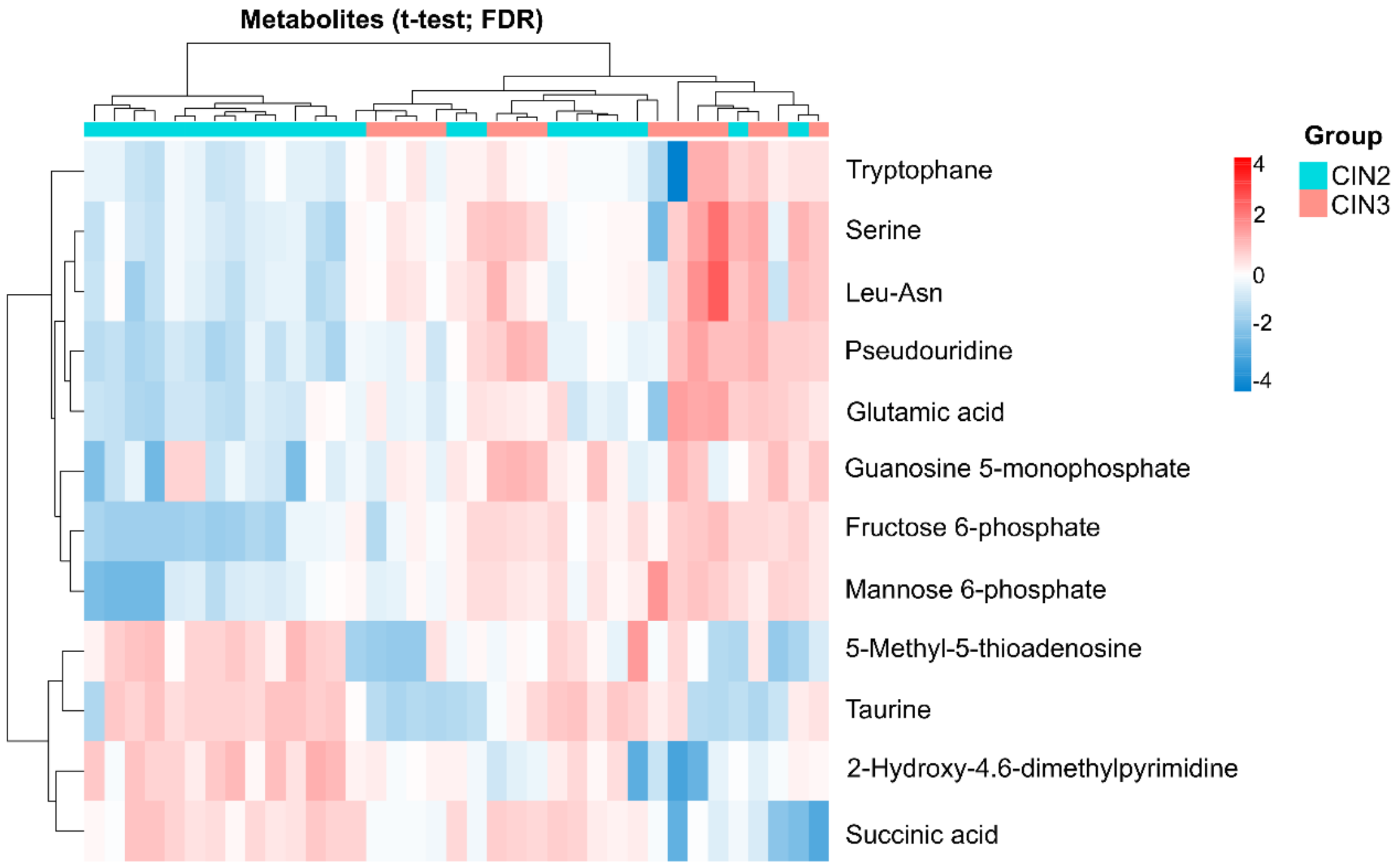

Figure S4- Heatmap of metabolites meeting the predefined univariate significance criteria (p-value < 0.05 and false discovery rate [FDR] < 0.05). Color intensity represents normalized and scaled relative abundance across samples.

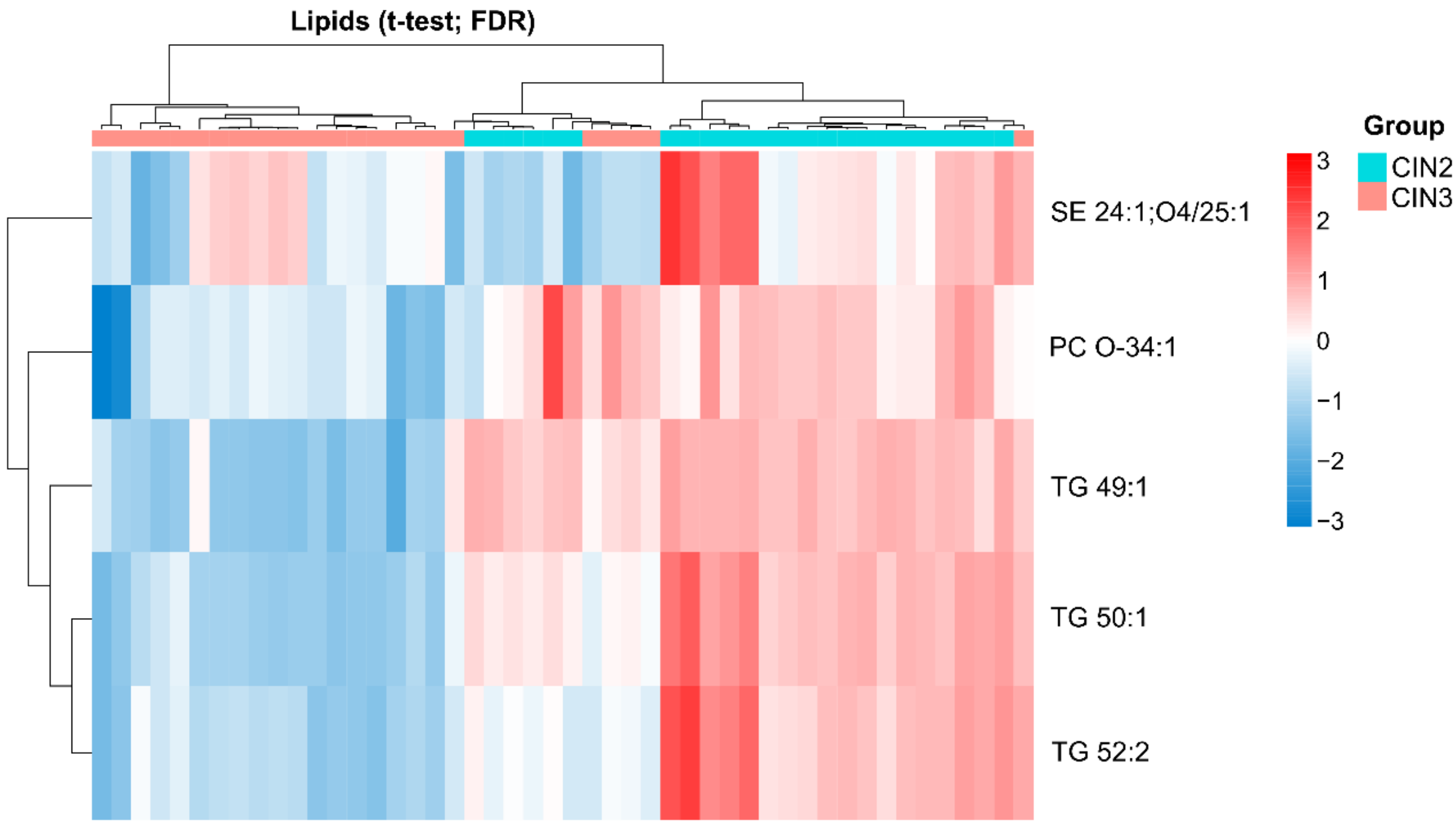

Figure S5- Heatmap of lipids meeting the predefined univariate significance criteria (p-value < 0.05 and false discovery rate [FDR] < 0.05). Color intensity represents normalized and scaled relative abundance across samples.

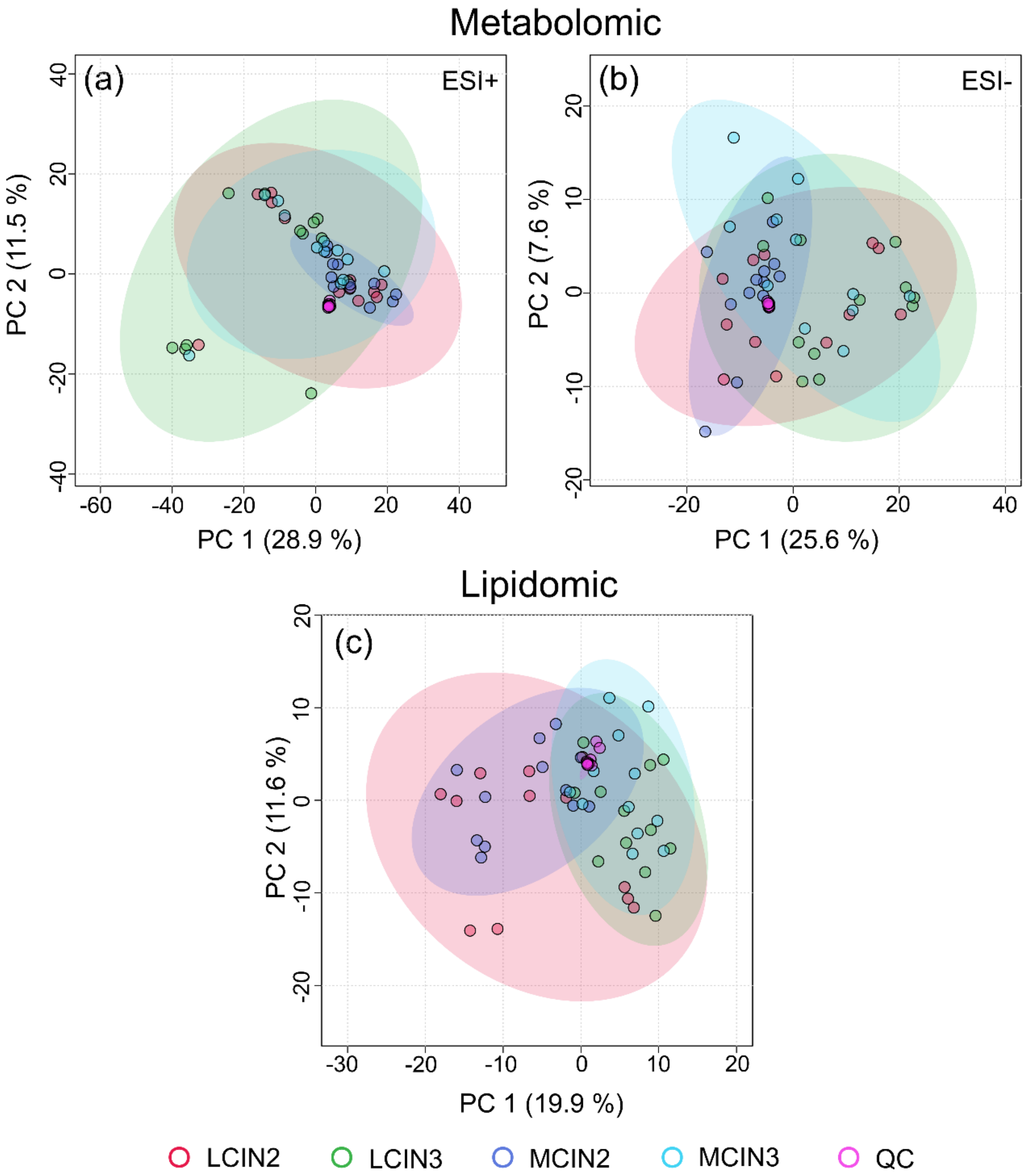

Figure S6 – PCA samples CIN 2 – lesion (LCIN2: red) e margin (MCIN2: dark blue) and CIN 3 – lesion (LCIN3: green) e margin (MCIN3: light blue) of the metabolomic (a and b) and lipidomic (c) study and quality control (QC: pink).
